# Downregulation of NONO Suppresses Proliferation, Migration, and Invasion in Metastatic Prostate Cancer

**DOI:** 10.1002/pros.24925

**Published:** 2025-06-12

**Authors:** Anna‐Lena Lemster, Sarah Grünhagen, Sarah Schmalfeld, Florian Lenz, Anna Natzius, Anne Offermann, Sven Perner, Jiong Zhang, Verena Sailer, Jutta Kirfel

**Affiliations:** ^1^ Institute of Pathology University Hospital Schleswig‐Holstein Lübeck Germany; ^2^ Gerhard‐Domagk Institute of Pathology University Hospital Münster Münster Germany; ^3^ Center for Outpatient Oncology Tübingen Tübingen Germany

**Keywords:** invasion, migration, NONO, PC3, proliferation, prostate cancer

## Abstract

**Background:**

As prostate cancer (PCa) remains one of the leading causes of cancer‐related death in men, it is important to develop effective therapeutic approaches for the treatment of metastatic PCa and to improve the accuracy of prognosis for aggressive tumors. Non‐POU domain‐containing octamer‐binding (NONO) is involved in RNA transport and almost all steps of gene regulation, and aberrant expression is associated with tumorigenesis in various tumor entities.

**Methods:**

Immunohistochemical analysis of the expression of NONO was performed in a cohort of 405 patient samples, including 54 benign prostate tissues, 250 primary prostate tumors, and 101 metastatic tissue samples. To assess the role of NONO in PCa cells, siRNA‐mediated knockdown of NONO in the metastatic PCa cell lines PC3 and DU145, followed by a series of functional assays including proliferation, transwell, western blotting, and real‐time quantitative PCR, was used.

**Results:**

The current study showed that the nuclear expression of NONO increases during the progression of PCa and is highest in distant metastases. NONO regulates cell proliferation by increasing the expression of cell cycle‐related genes. In addition, NONO modulates migration and invasion in PCa cells.

**Conclusion:**

Overall, these results suggest inhibition of NONO may be a promising approach for the treatment of metastatic PCa.

## 1 Introduction

Prostate cancer (PCa) is the most frequent type of cancer in the European male population (20.0% of all cancer cases in Europe) and it is the second most common cancer in men worldwide. It is the third most common cause of cancer‐related deaths in Europe [1]. A total of 10%–20% of PCa patients develop castration‐resistant prostate cancer (CRPC) within 5 years, which is characterized by disease progression after surgical or medical castration (androgen deprivation). Compared to hormone‐sensitive PCa, patients with CRPC have an increased risk of bone metastases and a significantly reduced survival time of only 14 months from the time of CRPC diagnosis [2].

Currently, the World Health Organization (WHO) grade grouping based on architectural features and the measurement of serum prostate‐specific antigen (PSA), along with other clinical parameters such as T‐stage, is primarily used to predict the prognosis and progression of PCa. However, the PSA value in particular shows clear limitations due to its poor sensitivity, specificity, and predictive value [3, 4], and the grade grouping also causes uncertainty in risk stratification [5]. Androgen deprivation therapy (ADT) with drugs altering the androgen receptor axis and conventional chemotherapy are still the most frequently used therapeutic approaches in several clinical settings [6]. Actionable genetic alterations such as microsatellite instability and BRCA mutations are found in a subset of patients and enable immunotherapy and targeted therapy [6]. However, these improvements have not resulted in the development of effective therapeutic approaches for the majority of PCa patients so far. Finding new predictive biomarkers is therefore very important. In recent studies, the non‐POU domain‐containing octamer‐binding (NONO) protein has emerged as a potentially relevant biomarker in other cancer types [7, 8]. NONO is a 54 kDa protein of the Drosophila behavior/human splicing (DBHS) protein family. NONO consists of tandem N‐terminal RNA recognition motifs (RRM1 and RRM2), a NONA/paraspeckle (NOPS) domain, a C‐terminal coiled coil, as well as N‐ and C‐terminal intrinsically disordered domains of low complexity [9]. NONO exists as a homodimer [10] or as a heterodimer in combination with other proteins of the DBHS family [11]. NONO is primarily found in the nucleus and is located in the nucleoplasm, within microscopically visible subnuclear bodies, termed paraspeckles, or large complexes such as DNA repair foci [12, 13], but is also localized in the cytoplasm [14]. NONO binds to DNA and RNA and acts as a “molecular scaffold” interacting with a variety of proteins [15, 16]. In addition, NONO has been reported to promote phase separation to form condensates, e.g., paraspeckles [12]. As a result, NONO is involved in almost all steps of gene regulation, including mRNA splicing, DNA unwinding, regulation of transcription, and nuclear retention of defective RNA [9, 13]. Furthermore, NONO has been identified in RNA transport granules in the cell nucleus and cytoplasm and might be involved in protein synthesis processes [14].

Through its involvement in multiple mechanisms, NONO participates in numerous biological processes. It is an important regulator in cell proliferation, apoptosis, and migration [9, 17, 18]. NONO is also involved in the early stages of DNA damage response, laying the foundation for double‐strand break repair [19]. Due to its involvement in these biological mechanisms, dysregulation of NONO leads to various pathological conditions. NONO overexpression is correlated with several types of cancers, including esophageal squamous cell carcinoma [18], lung [8], colorectal [17], and breast cancer [7]. Interestingly, in breast cancer, estrogen receptor (ER) negative cells generally show a reduced NONO expression compared to ER‐positive subtypes [7].

The role of NONO in PCa, however, has not yet been extensively studied. Therefore, the aim of this study was to characterize the role of NONO in PCa. We assessed clinical relevance in an immunohistochemically stained PCa cohort and molecular mechanisms of NONO in the PCa cell line PC3. In our clinicopathological study, NONO expression correlated only with tumor progression. We were able to show that NONO expression contributes to cell proliferation in PC3 and DU145 cells, which might be facilitated by cell cycle promotion. The NONO expression also promotes invasion and migration of these tumor cells.

## 2 Materials and Methods

### 2.1 Patient Data, Tumor Material, Immunohistochemical Analysis, and Quantification of Protein Expression

The study (project code: 17‐313) was conducted in accordance with the Declaration of Helsinki and approved by the Ethics Committee of the University of Lübeck. As the study is retrospective with anonymized clinical‐pathological data, informed consent of the patient was waived. Tissue samples and clinicopathological data of 405 patients were provided by the Institute of Pathology of the University Hospital Schleswig‐Holstein, Campus Lübeck, and the Institute of Pathology of the Klinik am Eichert, Alb Fils Klinikum, Göppingen. The cohort consists of 54 benign tissue, 250 primary tumors, and 101 metastatic tissue samples (Supporting Information S1: Table S1). A possible correlation of nuclear NONO expression with clinicopathological data was investigated using 232 primary tumor samples (Supporting Information S1: Table S2).

Tissue microarray construction and immunohistochemical analysis were performed as previously described [20]. For immunohistochemical analysis, the Ventana BenchMark ULTRA automated staining system (Roche, Basel, Switzerland), the Ventana OptiView DAB IHC Detection Kit (Roch), and Ventana Cell Conditioning Solution 2 (#950‐123, Roche) were used. The polyclonal anti‐NONO antibody (catalog nr. HPA054094, lot nr. R72098, RRID AB_2682376, Sigma Aldrich, St. Louis, MO, USA) was used as the primary antibody at a dilution of 1:250. Sections were digitized using the Ventana DP 200 Slide Scanner (Ventana Medical System) and analyzed using QuPath (v0.4.0, Edinburgh, Scotland) as previously described [20].

### 2.2 Cell Culture and siRNA Transfection

PC‐3 (RRID:CVCL_0035, CLS Cell Lines Service, Eppelheim, Germany) cells were cultured with DMEM/F12 medium, LNCaP (RRID:CVCL_0395, Leibniz Institute DSMZ, Braunschweig, Germany) and DU145 (RRID:CVCL_0105, Leibniz Institute DSMZ) cells were cultured with RPMI 1640 medium. All culture media were supplemented with 10% FBS (Biowest, Riverside, MO, USA) and 1% penicillin/streptomycin, culture media for PC3 and LNCaP were also supplemented with 1% l‐glutamine (Thermo Fisher). All human cell lines have been authenticated using STR profiling within the last 3 years, and all experiments were performed with mycoplasma‐free cells.

For the reverse transfection of the siRNA, a pool of three different Silencer Select siRNAs for NONO was used to reduce off‐target effects [21]. A total of 135 pmol of siRNA pool for NONO (439240: ID s9612, ID s9613, ID s9614) or 135 pmol of the Silencer Select Negative Control Nr. 2 siRNA (4390846) and 6 µL of Lipofectamine RNAiMAX transfection reagent (13778100) were diluted in a 6‐well plate with a final volume of 500 µL OptiMEM and incubated for 20 min. A total of 3 × 10^5^ cells/well were diluted in 2.5 mL Medium and added to the siRNA‐lipid complex. The cells were incubated for 48 h in DMEM/F12 medium supplemented with 10% FCS and 1% l‐glutamine. Following transfection, the cells were used for various experiments. Unless otherwise stated, materials used were purchased from Thermo Fisher Scientific, Waltham, MA, USA.

### 2.3 Proliferation Monitoring

Following transfection, the cells were cultured and then detached at various time points (0, 24, 48, and 72 h). The cell suspension was then stained 1:1 with trypan blue 0.4% solution (Logos Biosystems Inc., Anyang, South Korea). The number of living cells/mL was counted in triplicate using the PhotonSlide (Logos Biosystems Inc., Anyang, South Korea) for the LUNA‐FL Dual Fluorescence Cell Counter (BioCat GmbH, Heidelberg, Germany) in brightfield mode.

### 2.4 Migration and Invasion Assay

The motility of untreated and transfected cells was analyzed by transwell migration (Transwell‐Boyden chamber, #353097) and invasion chamber assays (BioCoat Matrigel Invasion chamber, #354480, both from Corning Life Sciences, Tewksbury, MA, USA). For both assays, 6 × 10^4^ cells were added with 2% FBS medium in the upper chamber of 24‐well inserts. Medium containing 10% FBS was added to the lower chamber. The cells were incubated for 24 h. Cells were fixed with 4% paraformaldehyde (neoFroxx, Einhausen, Germany) for 10 min, washed with PBS and ddH_2_0, and stained with Harris' hematoxylin (Th. Geyer, Renningen, Germany) for 20 min. The total number of cells was counted. After removing the non‐migrated or non‐invaded cells from the upper chamber, membranes were removed from the plastic holders, mounted on glass slides with Faramount Aqueous Mounting Medium Ready‐to‐use (Dako, Agilent Technologies, Santa Clara, CA, USA), and the number of migrated or invaded cells was counted. The total number of cells and migrated or invaded cells were counted in five ×20 magnification fields per assay. The assays were performed in duplicates.

### 2.5 RNA Isolation and Quantitative Real‐Time PCR (qRT‐PCR)

RNA was extracted using RNeasy mini kit with an on‐column DNase digestion with the RNase‐free DNase set (both from Qiagen, Venlo, Netherlands) following the manufacturer's protocol. The cDNA synthesis was performed with 1 µg RNA using the High‐Capacity RNA‐to‐cDNA Kit (Thermo Fisher Scientific). Gene expression was monitored by qRT‐PCR using the LightCycler 480 and the LightCycler 480 SYBR Green I Master Kit (both from Roche Holding, Basel, Switzerland). Each sample was run in triplicate. The relative expression was determined by normalization to the mean cycle threshold of the reference genes, TATA‐binding protein (*TBP*), Hypoxanthine‐Guanine Phosphoribosyl Transferase (*HPRT*), and β‐actin (extended ΔCT‐method as described in Riedel et al. [22]), followed by normalization to shControl using the 2^−ΔΔCt^ method. Primer sequences are available in the Supporting Information Materials (Supporting Information S1: Table S3).

For RT^2^ Profiler PCR arrays, cDNA was synthesized from 400 ng RNA using the RT^2^ First Strand Kit. Gene expression was monitored on the RT^2^ Profiler PCR array human epithelial‐to‐mesenchymal transition (EMT) 384‐well plate (GeneGlobe ID PAHS‐090ZG‐4) or the RT^2^ Profiler PCR array human cell cycle 384‐well plate (GeneGlobe ID PAHS‐020ZG‐4) using the RT^2^ SYBR Green qPCR Mastermix according to the manufacturer's protocol (all from Qiagen, Venlo, Netherlands). Gene expression measurement was performed on the LightCycler 480. The relative expression was determined by normalization to the reference genes *ACTB*, *B2M*, *GAPDH*, *HPRT1*, and RPLP0 followed by normalization to shControl as described above.

### 2.6 Protein Extraction and Western Blot Analysis

Proteins were extracted using Pierce RIPA buffer with added protease and phosphatase inhibitors (protease inhibitor cocktail, phosphatase inhibitor cocktail C2, and phosphatase inhibitor cocktail C3; all from Sigma Aldrich). The protein concentration was determined using the Pierce BCA protein assay kit. Sodium dodecyl sulfate‐polyacrylamide gel electrophoresis (SDS‐PAGE) and semi‐wet blotting were performed using the NuPAGE electrophoresis system (Thermo Fisher Scientific) with 4%–12% Bis‐Tris gels and PVDF membranes under reducing conditions according to manufacturer's protocol. A total of 30 µg or 60 µg (for SNAIL and the corresponding β‐actin loading control) of protein was loaded onto the gel. The membranes were blocked in 5% milk in Tris‐buffered saline with Tween20 (TBS‐T; 20 mM Tris‐HCl, 1369 mM NaCl, 0.075% Tween20, pH 7.5) for 1 h at room temperature. Membranes were cut and incubated with the different primary antibodies overnight at 4°C. Membranes were washed in TBS‐T three times for 8 min each and incubated with secondary antibodies for 1 h at room temperature. Membranes were rinsed three times with demineralized water and washed with TBS‐T for 10 min. Membranes were incubated with Pierce ECL Western blotting substrate for 5 min and developed using the Amersham Imager 600 (GE Healthcare Bio‐Sciences, Uppsala, Sweden). Unless otherwise stated, materials used were purchased from Thermo Fisher Scientific. The antibodies used in this study can be found in the supporting materials (Supporting Information S1: Table S4). Image StudioTM Lite Ver 5.2 (LI‐COR Biosciences, Licoln, NE, USA) was used for quantitative analysis of the band intensity. The background signal above and below the band was subtracted from each individual band. The normalization factor was calculated by dividing the intensity of each β‐actin band by the highest loading control signal detected on the blot. Each target band signal was divided by the lane normalization factor [23].

### 2.7 Statistical Analysis

Statistical analysis of immunohistochemical data were performed using jamovi (v2.4.11, The jamovi Project [2024], https://www.jamovi.org, accessed on December 12, 2023) and the R packages finalfit (Harrison E and Drake T, Ots R [2019] *finalfit: Quickly Create Elegant Regression Results Tables and Plots when Modelling*. https://cran.r-project.org/package=finalfit, accessed on December 12, 2023) and survival (Therneau T [2020]. *A Package for Survival Analysis in R*. https://cran.r-project.org/package=survival, accessed on accessed on December 12, 2023). As variance homogeneity was violated within the cohort, Welch's ANOVA and the Games‐Howell post hoc test were used to calculate differences in nuclear NONO expression in the different tissue types. For the statistical analysis of nuclear NONO expression with the clinicopathological data, the condition of variance homogeneity in the cohort was fulfilled, and consequently, the student's *t*‐test or, in case of more than two variables, Fisher's ANOVA with the Tukey post hoc test was used. Boxplots show the 25th to 75th percentiles, all data points, and the minimum and maximum as whiskers. For survival analysis, the mean value of nuclear NONO expression was used as a cut‐off value to divide the cohort into groups with high and low NONO expression. The Kaplan–Meier method was used to analyze the 5‐year progression‐free survival, and the log‐rank test was used for statistical analysis.

Data obtained from cell culture were analyzed using GraphPad Prism 10 V10.2.3 (GraphPad Software, Boston, MA, USA). Homogeneity in variance as evaluated by the D'Agostino‐Pearson test. If homogeneity was violated, the Kruskal–Wallis test was used for statistical analyses; if homogeneity was not violated, one‐way or two‐way ANOVA tests were used depending on the number of variables. Error bars indicate the 95% confidence interval (CI) unless otherwise stated.

## 3 Results

### 3.1 Elevated NONO Expression in Primary PCa and Metastases

**Figure 1:**
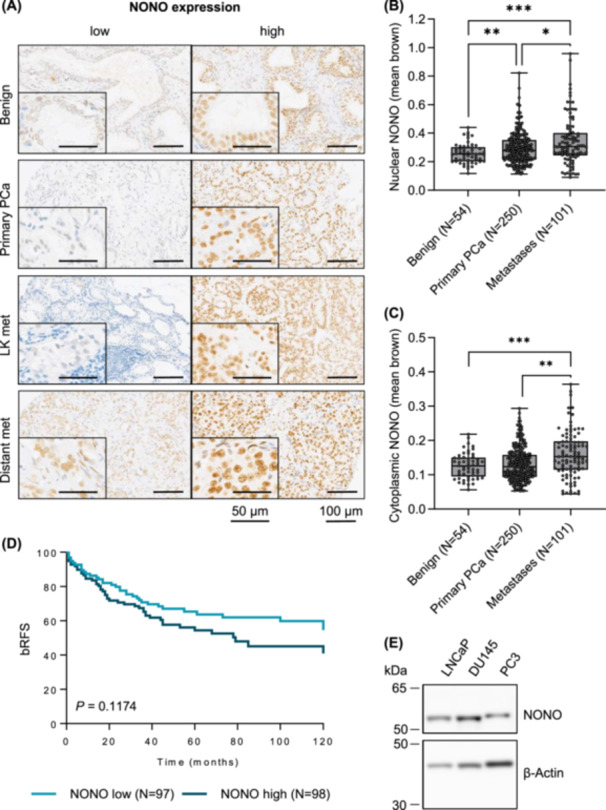
Non‐POU domain‐containing octamer binding (NONO) is overexpressed in primary tumors and metastases of prostate cancer. (A) Representative immunohistochemistry images show low and high NONO expression in benign tissue, primary prostate cancer (PCa), lymph node metastases (LK met), and distant metastases (Distant met). Nuclear (B) and cytoplasmic NONO (C) expression in our cohort was higher in primary PCa and metastases than in benign tissue. *p*‐values were calculated using Welch's ANOVA and the Games‐Howell post hoc test. (D) Kaplan–Meier plot shows no difference in 5‐year biochemical recurrence‐free survival (bRFS) of patients with high versus low nuclear NONO expression in primary PCa. The cohort was categorized into two groups based on the median expression of NONO. The expression above or equal to the median and expression below the median were considered high and low, respectively. *p‐*value was calculated using the two‐sided log‐rank test. (E) The NONO protein level was determined in the PCa metastasis cell lines LNCaP, DU145, and PC3 using western blotting. β‐Actin was used as a loading control (*n* = 4). * = *p* < 0.05, ** = *p* < 0.01, *** = *p* < 0.001. [Color figure can be viewed at wileyonlinelibrary.com]

To assess the clinical significance of NONO in our PCa cohort, an immunohistochemical analysis of NONO was performed. Each distinct PCa focus of the cohort was represented by at least one triplet of cores on a TMA. For the statistical analysis, the mean value of all foci triplets from each patient was used. The overall intensity of NONO protein staining in the cohort was variable and ranged from negative to strongly positive in both benign and malignant tissue. However, the NONO staining intensity within a single core and within the patient triplet was homogeneous. NONO expression was found in both the nucleus and cytoplasm, with overall stronger expression in the nucleus than in the cytoplasm (Figure 1A–C). The nuclear expression significantly correlated with the cytoplasmic expression (*p* < 0.0001, Supporting Information S1: Figure S1). In several cells, punctate NONO staining was observed, which likely indicates paraspeckles formation (Supporting Information S1: Figure S2).

First, the intensity of nuclear NONO staining (mean brown) was compared in 54 benign tissue samples, 250 primary tumors, and 101 metastases. The nuclear NONO expression was significantly lower in benign tissue than in primary tumor (*p* = 0.008) and metastatic tissue samples (*p* < 0.001). In addition, the nuclear expression of NONO was found to be significantly higher in metastatic tissue than in primary tumor tissue (Figure 1B, *p* = 0.024). Similar results were observed when comparing the cytoplasmic expression of NONO in the different tissue types (Figure 1C), although the difference in cytoplasmic expression of NONO in benign tissue and primary PCa was not significant (*p* = 0.281). When the metastatic tissue was further subdivided into lymph node metastases and distant metastases, distant metastases showed higher nuclear NONO expression than lymph node metastases, however, the difference was marginally non‐significant (Supporting Information S1: Figure S2A, *p* = 0.058).

Next, a correlation between nuclear NONO expression and clinicopathological data of the patients was investigated. Tumors from patients who had received hormone therapy showed slightly increased, although not significant, nuclear NONO expression compared to tumors from patients who did not receive hormone therapy, with higher expression in patients who had received hormone therapy before the surgery in which the tissue was harvested (*p* = 0.228; Supporting Information S1: Figure S3B) than in patients who were treated after the surgery (*p* = 0.522). The analysis of NONO expression in relation to other clinicopathologic data (grade group, T‐stage, preoperative PSA level of the patient, N status, and R status), however, showed no discernible trends (Supporting Information S1: Figure S3C,G).

The 10‐year biochemical recurrence‐free survival (bRFS) analysis shows that patients with high nuclear NONO expression had a slightly lower, although not significant, bRFS than patients with low NONO expression (Figure 1D, *p* = 0.117). While 34.6% of patients with low NONO expression developed a PCa recurrence after 5 years and 45.5% of patients developed a recurrence after 10 years, this figure increased in patients with high NONO expression to 45.6% recurrence after 5 years and 59.0% after 10 years.

To perform functional studies, the expression of NONO in cell lines isolated from PCa lymph node metastasis (LNCaP), brain metastasis (DU145), and bone metastasis (PC3) was characterized. LNCaP, DU145, and PC3 cells showed comparable NONO protein levels (Figure 1E and Supporting Information S1: Figure S4A,B). Previous studies show that the metastatic potential increases from LNCaP to DU145 to PC3 cells and is much larger in PC3 cells compared to both LNCaP and DU145 cells (also see Supporting Information S1: Figure S4C for E‐cadherin and N‐cadherin expression in these cell lines) [24]. Since a major challenge in the treatment of PCa is the treatment of highly aggressive and metastatic PCa that does not respond to hormone therapy, the role of NONO expression was further investigated in NONO high‐expressing, metastatic but androgen‐insensitive DU145 and PC3 cells.

### 3.2 NONO Knockdown Represses PCa Cell Proliferation

**Figure 2:**
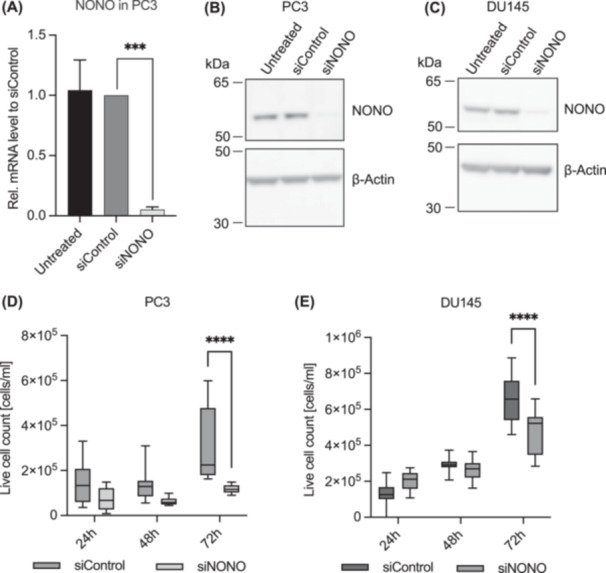
Non‐POU domain‐containing octamer binding (NONO) is associated with cell proliferation in prostate cancer cells. (A) Knockdown efficiency of siRNAs targeting NONO in PC3 cells was confirmed by qRT‐PCR. mRNA levels were determined by normalization to the mean of TBP, HPRT, and β‐actin and presented as mean fold change ± 95% CI to siControl (*n* = 7). Reduced protein expression of NONO after siRNA transfection in PC3 (B) and DU145 (C) cells was analyzed by Western blotting. β‐Actin was used as a loading control (*n* = 9 for PC3 transfection and *n* = 3 for DU145 transfection). Inhibitory effects of siRNAs targeting NONO on the cell proliferation of PC3 (D) and DU145 (E) cells were analyzed by the cell count at different time points (24, 48, and 72 h) following transfection. Results are shown as live cells/mL with the minimum and maximum as whiskers (*n* = 3). *** = *p* < 0.001, **** = *p* < 0.0001.

Since primary PCa and metastases showed increased NONO expression compared to benign prostate tissue, we next investigated whether NONO contributes to cell proliferation in PCa. SiRNA transfection with a pool of three different siRNAs targeting NONO in PC3 and DU145 cells was used to knockdown the expression of NONO. The transfection was able to significantly reduce NONO expression in PC3 cells at both mRNA (Figure 2A, *p* = 0.0001) and protein levels (*p* = 0.0006, Figure 2B, see Supporting Information S1: Figure S5A,B for original Western blot and quantification). The mRNA level in PC3‐NONO knockdown cells (siNONO) was reduced by 0.95‐fold compared to the siRNA‐transfected control cells (siControl). The expression of NONO was also successfully reduced in DU145 cells (Figure 2C and Supporting Information S1: Figure S5C,D).

We monitored the proliferation of PC3 and DU145 cells after siRNA transfection targeting NONO by counting live cells over the course of 72 h. The knockdown of NONO was able to significantly reduce the proliferation in PC3 (Figure 2D, *p* < 0.0001) and DU145 (Figure 2E, *p* < 0.0001) cells compared to siRNA control cells. The regulating effect NONO has on proliferation in PC3 cells was verified using the MTT cell proliferation assay (Supporting Information S1: Figure S6A). Reduced cell proliferation of siControl cells in the MTT assay might be due to off‐target effects that unfortunately always occur in siRNA transfections and that often have reproducible growth‐restricting effects on mammalian cells in culture [25], but even compared to siControl cells, siNONO cell proliferation was significantly reduced. Therefore, the reduction in cell proliferation in siNONO cells is, at least partially, caused by knockdown of NONO. To ensure the NONO knockdown was stable for 72 h, the protein level of NONO was determined at different time points during the cell proliferation assay. The NONO protein level remained unchanged in siRNA‐transfected cells (Supporting Information S1: Figure S6B,C).

**Figure 3:**
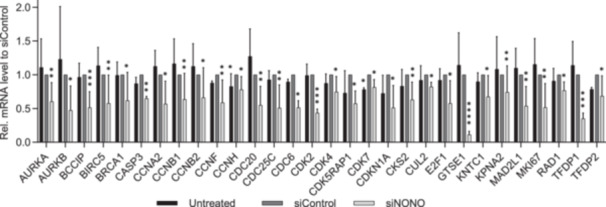
Significant downregulation of genes related to cell cycle after knockdown of non‐POU domain‐containing octamer binding (NONO). Differences in gene expression after siRNA transfection in PC3 cells targeting NONO was analyzed by RT^2^ Profiler PCR array for human cell cycle genes. mRNA levels were determined by normalization to the mean of ACTB, B2M, GAPDH, HPRT1, and RPLP0 and presented as mean fold change ± 95% CI to siControl (*n* = 4). * = *p* < 0.05, ** = *p* < 0.01, *** = *p* < 0.001, **** = *p* < 0.0001.

Since NONO regulated proliferation in PC3 cells, we investigated the effect of NONO on the expression of cell cycle genes. Of the 82 genes analyzed using the RT^2^ Profiler PCR array for the human cell cycle (Supporting Information S1: Table S5), no genes were significantly upregulated, whereas 30 genes were significantly downregulated (Figure 3). The expression of the marker of proliferation Ki‐67 (*MKI67*) was reduced 0.52‐fold in the NONO knockdown cells compared to the siRNA‐transfected control. 10 significantly reduced genes are associated with the G2 phase and G2/M transition (*BCCIP*, *BIRC5*, *CCNA2*, *CCNB1*, CCNH, *CDK5RAP1*, *CDK7*, *CKS2*, *GTSE1*, and *KPNA2*), while 6 genes are associated with the M phase (*AURKB*, *CCNB2*, *CCNF*, *CDC20*, *CDC25C*, and *CDC6*). Interestingly, although cell proliferation and cell cycle gene expression were significantly reduced in NONO knockdown cells, knockdown did not affect the protein level of the cell proliferation marker proliferating cell nuclear antigen (PCNA; Supporting Information S1: Figure S7).

We also investigated whether NONO played a role in apoptosis or autophagy in PC3 cells. All cells showed reduced apoptosis and autophagy behavior 72 h after siRNA transfection, but no differences could be observed between NONO knockdown cells and control cells. Therefore, both apoptosis and autophagy were not altered after NONO knockdown (Supporting Information S1: Figure S8).

### 3.3 NONO Knockdown Reduces Migration and Invasion in PCa Cells

**Figure 4:**
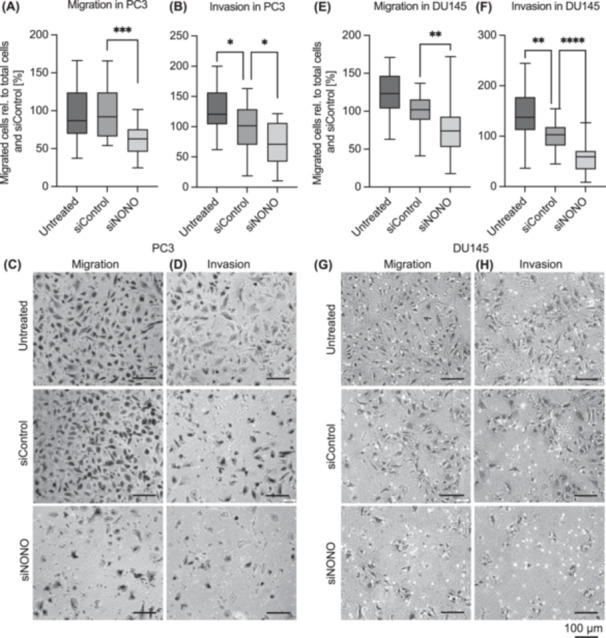
Knockdown of non‐POU domain‐containing octamer binding (NONO) reduces migration and invasion in prostate cancer cells. Transfection with siRNAs targeting NONO significantly reduced the migration (A and C) and invasion (B and D) ability of PC3 cells, as well as the migration (E and G) and invasion (F and H) ability of DU145 cells. This was analyzed by transwell migration and invasion chamber assays. Boxplots show the data as a total number of cells and to siControl at 100% with the 25th to 75th percentiles and the minimum and maximum as whiskers (*n* = 3). A representative result of the cell migration and invasion assay is shown. * = *p* < 0.05, ** = *p* < 0.01, *** = *p* < 0.001, **** = *p* < 0.0001.

To examine the role of NONO in metastatic processes, we performed in vitro migration and invasion assays. Knockdown of NONO significantly reduced the migration and invasion by 37.5% (Figure 4A,C, *p* = 0.0001) and 30.8% (Figure 4B,D, *p* = 0.0205), respectively, compared to the siRNA‐transfected control. In Du145 cells, NONO knockdown also reduced the migration by 24.4% (Figure 4E,G, *p =* 0.0034) and the invasion by 43.12% (Figure 4F,H, *p* < 0.0001) compared to siControl cells.

**Figure 5:**
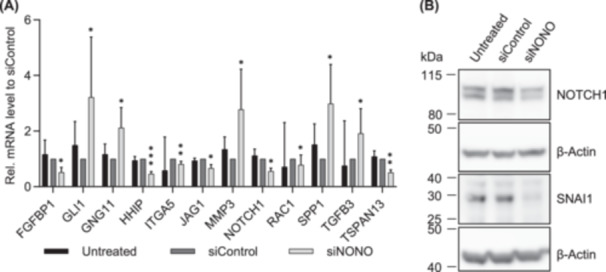
Slight changes of gene and protein expression associated with epithelial‐to‐mesenchymal transition (EMT) after knockdown of non‐POU domain‐containing octamer binding (NONO) in prostate cancer cells. (A) Differences in expression of EMT‐associated genes after siRNA transfection in PC3 cells targeting NONO were analyzed by qRT‐PCR and RT^2^ Profiler PCR array for human EMT genes (for ITGA5, RAC1, and TGFB3). mRNA levels were determined by normalization to the mean of TBP, HPRT, and β‐actin or the mean of ACTB, B2M, GAPDH, HPRT1, and RPLP0 (for ITGA5, RACK1, and TGFB3) and presented as mean fold change ± 95% CI to siControl (for JAG1 and NOTCH1 *n* = 6; for FGFBP1, GLI1, GNG11, MMP3, SPP1, and TSPAN13 *n* = 5; for HHIP *n* = 4; for ITGA5, RAC1, and TGFB3 *n* = 3). (B) Reduced protein level of NOTCH1 and SNAI1 after knockdown of NONO in PC3 cells was analyzed using Western blotting. β‐Actin was used as a loading control (*n* = 6). * = *p* < 0.05, ** = *p* < 0.01, *** = *p* < 0.001.

We analyzed the gene expression and protein level of genes associated with the EMT. Using qRT‐PCR and the RT^2^ Profiler PCR array for human EMT, the expression of 90 different EMT‐related genes was analyzed in NONO knockdown and control cells (Supporting Information S1: Table S6). Only 12 of these genes were significantly affected by NONO knockdown. The expression of *GLI1*, *GNG11*, *MMP3*, *SPP1*, and *TGFB3* was significantly increased, while the expression of *HHIP*, *FGFBP1*, *ITGA5*, *JAG1*, *NOTCH1*, *RAC1*, and *TSPAN13* was significantly decreased in the NONO knockdown cells (Figure 5A). NOTCH1 expression was also reduced at the protein level in the NONO knockdown cells compared to the control cells (Figure 5B and Supporting Information S1: Figure S9).

Interestingly, even though the migration and invasion were reduced after NONO knockdown and some EMT‐related genes were significantly differently expressed, the mRNA expression of E‐Cadherin (*CDH1*), N‐Cadherin (*CDH2*), Snail Family Transcriptional Repressor 1 (*SNAI1*), and *SNAI2* did not show differences compared to the control cells (Supporting Information S1: Figure S10A). At the protein level, SNAI1, however, was significantly reduced in the NONO knockdown cells compared to the siRNA‐transfected control cells (*p* = 0.0346, Figure 5B and Supporting Information S1: Figure S10B,C). In accordance, the protein level of E‐Cadherin, N‐Cadherin, and SNAI2 was not altered in NONO knockdown cells (Supporting Information S1: Figure S10B,C).

## 4 Discussion

PCa is a clinically variable disease ranging from indolent to widespread metastatic tumors. The major challenge is to identify features of the primary tumor that predict progressive disease and thus to separate patients who benefit from immediate and aggressive therapy from patients whose tumors will likely remain indolent. Metastatic and castration‐resistant PCa is characterized by a higher frequency of molecular alterations and expression differences in comparison to localized primary tumors. Therefore, biomarkers are a promising diagnostic and prognostic tool for PCa. However, so far, there are no routinely used biomarkers due to the lack of independence or reproducibility on independent cohorts, as well as low sensitivity and specificity. Previously, we found that increased lysine‐demethylase 5C (KDM5C) expression is associated with a reduced bRFS in PCa [26], and KDM5C knockdown significantly reduced EMT, migration, and invasion [27]. We could also show that KDM5C interacts with the DBHS proteins paraspeckle component 1 (PSPC1) and NONO in the PCa cell line PC3 [20]. Like KDM5C, PSPC1 is associated with a reduced bRFS in PCa in our cohort [20]. In the present study, we investigated the role of NONO in PCa, as PSPC1 frequently forms dimers with NONO.

In immunohistochemistry‐based analysis, we observed an increased expression of NONO in malignant primary PCa and especially in metastatic PCa compared to benign tissue. In addition, the expression of NONO was increased in distant metastases compared to lymph node metastases. These results confirm previous studies on the expression of NONO in PCa. Yamamoto et al. [28] and Takayama et al. [29] described an increased expression of NONO in localized PCa compared to benign tissue and an even higher expression of NONO in CRPC (results are based on the gene expression profiling (GSE35988) by Grasso et al. [30]). Increased NONO abundance also correlates with the disease progression in other malignant tumors like esophageal squamous cell carcinoma [18], lung [8], and breast cancer [7, 31]. In some cancers, the NONO expression level is an independent prognostic factor [31, 32]. Takayama et al. [29] reported a significantly lower cancer‐specific survival of PCa patients with high NONO‐expressing tumors compared to tumors with low NONO expression. In our cohort, we investigated the rate at which tumors developed biochemical recurrence after surgery. Patients with low NONO‐expressing tumors had a slightly higher bRFS rate than tumors with high expression, but this difference is only a trend. Interestingly, although NONO expression is significantly increased during the malignant progression of PCa, NONO expression in our cohort did not correlate with other clinicopathologic parameters such as T‐stage or PSA value.

Since NONO expression was particularly elevated in distant metastasis in our PCa cohort, we investigated the role of NONO in common molecular mechanisms underlying PCa progression. By knocking down NONO expression in PC3 cells, a cell line derived from bone metastasis of PCa, we examined the involvement of NONO in different cellular processes. Knockdown of NONO significantly reduced the proliferation of PC3 and DU145 cells. This is in accordance with the results of a previous study on the role of NONO in a cell line derived from LNCaP cells with androgen‐independent characteristics and osteoblastic phenotype (LNCaP‐SF). Downregulation of NONO significantly reduced the growth rate of LNCaP‐SF cells and the tumor growth of NONO knockdown LNCaP‐SF cells in mouse xenografts [28]. In our study, NONO modulated proliferation in PC3 cells through genes associated with the cell cycle progression. Among these genes, *MKI67*, which is expressed in all cycling cells except for cells in the G0 phase [33] was downregulated in NONO knockdown cells compared to the control. Several other studies have demonstrated a role for NONO in maintaining the cell cycle. NONO can bind to transcription factors to act as a transcriptional cofactor in the regulation of gene expression, such as cell cycle gene expression [34, 35]. In acute monocytic leukemia THP1 cells reduced expression of NONO leads to increased proliferation and an accelerated cell cycle, as the number of cells in the G1 phase decreases and the number of cells in the S and G2/M phase increases [36]. In the cervical cancer cell line HeLa and in breast cancer cell lines, on the other hand, knockdown of NONO reduces cell proliferation by delaying G1/S transition [31, 37]. In the current study, no genes involved in the cell cycle, which were analyzed, were significantly upregulated after the knockdown of NONO. NONO knockdown did not affect the expression of PCNA, a marker for G1/S phase [33], and only four genes related to G1 phase and the G1/S transition and the S phase and DNA replication (CDK4, CDC6, CUL2, and E2F) were downregulated after knockdown of NONO in PC3 cells. Interestingly, 10 genes associated with the G2 phase, and the G2/M transition, and 6 genes of the M phase were downregulated after the knockdown of NONO, suggesting that NONO may play a greater role in G2 and M phase in PCa. Most of the genes that are significantly downregulated after knockdown of NONO have already been described to play a notable or even a major role in PCa. Upregulation of, for example, AURKA, AURKB, BIRC5, CCNA2, CCNB1, CDK2, CDC20, CDC25C, GTSE1, or MKI67 correlated with poor prognosis of PCa patients and more aggressive PCa [38, 39, 40, 41, 42, 43, 44, 45, 46].

In esophageal squamous cell carcinoma, the downregulation of NONO led to a significant reduction in cell proliferation and induced apoptosis, thereby increasing cancer invasiveness [18]. In the present study, knockdown of NONO showed no effect on apoptotic cell death or autophagy in PC3 cells. This is consistent with a study in breast cancer where apoptosis was also not affected by NONO expression [31].

NONO influences migration and invasion in various types of cancers. In bladder cancer, NONO decreases cell migration and invasion [47], while it increases invasion in colorectal [17] and esophageal squamous cell carcinoma [18] and promotes migration and invasion in breast cancer [48]. In the current study, we showed a reduced invasion and a decreased migration in NONO knockdown PC3 and DU145 cells. Yamamoto et al. [28] also reported a decreased invasion after knockdown of NONO in LNCaP‐SF cells and hypothesized a reason for this could be alternative splicing of the growth factor receptor EPH Receptor A6 (EPHA6), which is induced by NONO knockdown. In the current study, fibroblast growth factor binding protein 1 (*FGFBP1*) was downregulated in the NONO knockdown cells, and transforming growth factor beta 3 (*TGFB3*) was upregulated. Both proteins are part of the growth factor signaling pathway and are reported to regulate the proliferation and invasion in various cells. FGFBP1 increases proliferation and invasion and promotes EMT in pancreatic cancer [49, 50]. TGFB3 induces EMT and promotes migration and invasion in PC3 cells, while it does not affect migration in LNCaP and DU145 cells. In DU145 cells, TGFB3 reduced proliferation, which in turn was not affected in PC3 cells [51]. However, in this study, the decreased migration, invasion, and proliferation in NONO knockdown cells are associated with increased TGFB3. The role of NONO in the regulation of other EMT‐related genes is ambivalent in PCa. Increased expression of GLI1, GNG11, MMP3, and SPP1 and decreased expression of HHIP in NONO knockdown cells suggest that downregulation of NONO expression might increase EMT in PC3 cells. Increased expression of GLI1 or decreased expression of HHIP, for example, as we observed in NONO knockdown cells, resulted in increased EMT in previous studies [52, 53]. In addition, upregulated expression of SPP1, again as observed in NONO knockdown cells, promoted EMT in CRPC via PI3K/Akt and Erk1/2 pathways in a previous study [54]. In contrast, decreased expression of NOTCH1, JAG1, ITGA5, RAC1, and TSPAN13 may suggest that downregulation of NONO expression decreases EMT in PC3 cells. NOTCH1 and JAG1 are both part of the Notch signaling pathway and participate in the EMT. NOTCH1 and JAG1 expression are associated with increased migration or invasion in PCa [55, 56], and both genes were downregulated in NONO knockdown cells. ITGA5 promotes EMT in squamous cell carcinoma cells [57], and knockdown of NONO led to reduced expression of ITGA5 in PCa. RAC1 and TSPAN13 have been shown to promote EMT [58, 59] and are both downregulated in NONO knockdown PCa cells.

While the gene expression of *SNAI1* and *SNAI2* was not altered between NONO knockdown and control cells in the current study, protein expression of SNAI1 was decreased after PC3 NONO knockdown cells. Transcriptional, translational, and degradational regulation, which fine‐tune protein abundance to the desired level, could be responsible for this difference in gene expression and protein abundance. SNAI1 increases paracrine cell proliferation in PCa cells and induces EMT by decreasing the expression of cell adhesion‐associated molecules like E‐cadherin and increasing mesenchymal markers such as vimentin [60]. EMT is not a binary state of epithelial or mesenchymal phenotype, but a gradual transition with various intermediate stages in between. Each EMT stage is associated with the expression of different genes, and one cancer entity may vary from another in terms of the genes expressed at each stage [61, 62]. Interestingly, in the current study, although the NONO knockdown cells showed reduced migration and invasion and a differential expression in some of the EMT‐associated genes like JAG1 and NOTCH1, this did not seem to influence the expression of the epithelial marker CDH1 (E‐cadherin) and the mesenchymal marker CDH2 (N‐cadherin). Previous studies described this phenomenon of increased migration but no change in expression of the EMT markers E‐cadherin and N‐cadherin in the collective migration of epithelial cells known as the unjammed transition, a mechanism distinct from the EMT [63].

Though the expression of NONO does not affect the expression of E‐cadherin and N‐cadherin, knockdown of NONO significantly reduces migration and invasion as well as the expression of several genes associated with the EMT as described above. To gain a better understanding of the genes and proteins affected by NONO knockdown and the role NONO plays in EMT, migration, and invasion, measurement of the total gene expression by RNA‐seq or measurement of protein expression profiles with mass spectrometry is needed.

## 5 Conclusions

Taken together, our results show that NONO plays an important role in the cell proliferation of PCa by regulating the expression of cell cycle genes and that NONO is involved in the migration and invasion of the cells. NONO could be a potential new therapeutic target for PCa, particularly in more advanced tumors, but further investigations are needed.

## Author Contributions

Conceptualization: Anna‐Lena Lemster and Jutta Kirfel. Methodology: Anna‐Lena Lemster and Jutta Kirfel. Validation: Anna‐Lena Lemster, Anna Natzius, Sarah Grünhagen. Formal analysis: Anna‐Lena Lemster and Sarah Grünhagen. Investigation: Anna‐Lena Lemster, Sarah Grünhagen, Anna Natzius, Sarah Schmalfeld, Jiong Zhang and Florian Lenz. Resources: Jutta Kirfel, Anne Offermann, and Sven Perner. Data curation: Anna‐Lena Lemster and Sarah Grünhagen. Writing – original draft preparation: Anna‐Lena Lemster and Sarah Grünhagen. Writing – review and editing: Anna‐Lena Lemster, Sarah Grünhagen, Sarah Schmalfeld, Jiong Zhang, Jutta Kirfel, Florian Lenz, Anne Offermann, Sven Perner, and Verena Sailer. Visualization: Anna‐Lena Lemster. Supervision: Jutta Kirfel. Project administration: Anna‐Lena Lemster and Jutta Kirfel. Funding acquisition: Jutta Kirfel. All authors have read and agreed to the published version of the manuscript.

## Ethics Statement

The study was conducted according to the guidelines of the Declaration of Helsinki and approved by the Ethics Committee of the University of Luebeck (17‐313).

## Consent

Patient consent was waived since only anonymized clinico‐pathological data were used retrospectively (approved by the Review Board of the University of Luebeck).

## Conflicts of Interest

The authors declare no conflicts of interest.

## Supporting information

Supporting Material Lemster et al.

## Data Availability

The data that support the findings of this study are available from the corresponding author upon reasonable request.
