# Supplementary material for: Downregulation of NONO Suppresses Proliferation, Migration, and Invasion in Metastatic Prostate Cancer
Source: Prostate. 2025 Jun 12;85(12):1121–33. doi: 10.1002/pros.24925 (PMC12278708; doi:10.1002/pros.24925)
Supplement: Supplementary file 1 — Supporting Material Lemster et al. [file PROS-85-1121-s001.pdf]

Supplementary Materials for

Downregulation of NONO suppresses proliferation, migration, and invasion in metastatic prostate cancer

Anna-Lena Lemster, Sarah Grünhagen, Sarah Schmalfeld, Florian Lenz, Anna Natzius, Anne Offermann, Sven Perner, Jiong Zhang, Verena Sailer, Jutta Kirfel

	Page
Supplementary Methods	
MTT Cell Proliferation Assay	2
Apoptosis and Autophagy Assay	2
Patient cohort Tables S1 to S2	2
Supplementary Materials Tables S3 to S4	3
Supplementary Results	
Figure S1	5
Figure S2	5
Figure S3	6
Figure S4	7
Figure S5	8
Figure S6	9
Table S5	9
Figure S6	10
Figure S7	11
Table S6	11
Figure S8	13
Figure S9	14

Supplementary Methods

MTT Cell Proliferation Assay

2000 untreated or transfected cells/well were added to a 96-well plate with 100 µl medium in 6-fold determination. The cells were cultured overnight to a confluence of 60-80%. The MTT assay was performed at different time points (0 h, 24 h, 48 h, and 72 h) with 10 µl thiazolyl blue tetrazolium bromide (MTT, Sigma Aldrich) and incubated for 3 h at 37°C.[22] Medium was removed and cells were incubated for 3 h at 37 °C with 100 µl MTT solvent (10% SDS from Carl Roth, Karlsruhe, Germany and 0.01 M HCl from Supelco®, Merck KGaA, Darmstadt, Germany). The absorbance was measured at 550 nm with a reference wavelength of 690 nm.

Apoptosis and Autophagy Assay

2,000 cells/100 µl medium were seeded in 6-fold determination in a 96-well plate. The cells were cultured overnight to a confluence of 90-100 %. For the apoptosis detection, the CellEvent™-Caspase-3/7 detection reagent (Thermo Fisher Scientific, Waltham, MA, USA) was diluted in PBS with 5 % FCS at a concentration of 5 µM. The medium was removed and 100 µl of detection reagent was added to each well. The 96-well plates were incubated for 40 min at 37 °C and measured at an absorbance/emission maximum of 502/530 nm. For the autophagy detection, the Autophagy Assay Kit (Merck, Darmstadt, Germany) was used according to the manufacturer's instructions with an incubation time of 40 min at 37 °C with the Autophagosome Detection Reagent Working Solution on the 96-well plates. The fluorescence intensity was measured at an absorbance/emission maximum of 360/520 nm.

Table S1.Cohort sorted by tissue type.

Characteristic	Category	N
Patients		405
Excluded		40
Tissue type	Benign	54
	Primary PCa	250
	Metastasis	101
	Lymph node metastases	24
	Distant metastases	19
	Unknown	58

Table S2. Characteristics of patients with primary tumor.

Characteristic	Category	N
Patients	Primary PCa	232
WHO Grade Group	G1	54
	G2	64
	G3	29
	G4	17
	G5	20
	Unknown	48
T stage	T1	14
	T2	104
	T3	62
	T4	25

Characteristic	Category	N
N stage	Unknown	27
	N0	198
	N1	19
	N2	1
R status	Unknown	14
	R0	147
	R1	66
	Unknown	19
PSA before surgery (iPSA, ng/ml)	Low (< 10ng/ml)	133
	Medium (10-20 ng/ml)	41
	High (> 20ng/ml)	39
	Unknown	19
Hormone therapy	No	152
	Yes	70
	Unknown	10
5-year biochemical recurrence-free survival	No recurrence	132
	Recurrence	90
	Unknown	10

Table S3. Primers used in the reverse transcription quantitative PCR in this publication.

Gene	Forward	Reverse
AXIN2	TGGCTATGTCTTTGCACCAG	TTCCATCTACACTGCTGTCCG
BPM7	GGGAACGCTTCGACAATGAG	AAGAGATCCGATTCCCTGCC
CDH1	AAGGGGTCTGTCATGGAAGG	GGTGTTCACATCATCGTCCG
CDH2	CCATCATTGCCATCCTGCTC	GTTTGGCCTGGCGTTCTTTA
COL1A2	GAGGGCAACAGCAGGTTCACTTA	TCAGCACCACCGATGTCCAA
COL3A1	TGGTCTGCAAGGAATGCCTGGA	TCTTTCCCTGGGACACCATCAG
ESR1	ATCCACCTGATGGCCAAG	GCTCCATGCCTTTGTTACTCA
F11R	GTGAAGTTGTCCTGTGCCTACTC	ACCAGTTGGCAAGAAGGTCACC
FGFBP1	CTTCACAGCAAAGTGGTCTCA	GACACAGGAAAATTCATGGTCCA
FN1	TAGCTTTGTGGTCTCCTGGG	AAGCAGGTCAGGGATGTTCA
FOXC2	CGGCCCAGCAGCAAACTTTCC	AGAGGCGGCGTGGATCTGTAG
GLI1	CAGGGAGGAAAGCAGACTGA	ACTGCTGCAGGATGACTGG
GNG11	CCTGCCCTTCACATCGAAGAT	CTTCTTTGCGAAGCTGCTCAA
HHIP	GCTCGCAACGTCTCTTCATT	GTCCTCTTTCATCTCCTCCCTT
IGFBP4	ACCCACGAGGACCTCTACATCA	CACACCAGCACTTGCCACGCT
IL1RN	ATGGAGGGAAGATGTGCCTGTC	GTCCTGCTTTCTGTTCTCGCTC
ITGA5	GCACCAACAAGAGAGCCAAA	CAGGATTCGGTAGGGCATCT
ITGAV	GCGGGACCATCTCATCACTA	CACTGAGCAACTCCACAACC
JAG1	GAATGGCAACAAAACTTGCAT	AGCCTTGTCGGCAAATAGC
KDM5C	GCCAACCTTGTGCAGTGTAA	TTGAACTTGGAAGGCTGCAC
KRT19	AGCTAGAGGTGAAGATCCGCGA	GCAGGACAATCCTGGAGTTCTC
KRT7	CATCGAGATCGCCACCTACC	GATATTCACGGCTCCCACTCC
MAP1B	CACCGAGGTGCGCTTAATGAT	GCTTAGTAACTCCCCGATCTCTT
MMP2	ATAACCTGGATGCCGTCGT	AGGCACCCTTGAAGAAGTAGC
MMP3	TGGCCATCTCTTCCTTCAGG	GGGTATCCAGCTCGTACCTC
NONO	AACCATACTCCAAGAAAGCATCA	GTGAAGGTCTTCTCTCCTGGTTT
NOTCH1	CAGCCTCAACATCCCCTACA	AGAACAGAAGCACAAAGGCG
OCLN	GCAAAGGGAAGAGCAGGAAG	GGATATTCCCTGATCCAGTCCT
RAC1	GGAGACGGAGCTGTAGGTAA	TAAGCCCAGATTCACCGGTT
SHH	ATGAAGAAAACACCGGAGCG	AGTTTCACTCCTGGCCACTG
SMAD2	CATCACAGCCCTCACTCACT	CGCACTCCTCTTCCTATATGC
SMAD4	ACTGGAAGTAGGACTGCACC	TGGAAATGGGAGGCTGGAAT
SOX10	TCATCCCTTCAATGCCCCCT	TGCGTCTCAAGGTCATGGAGG
SPARC	TGCCTGATGAGACAGAGGTGGT	CTTCGGTTTCCTCTGCACCATC
SPP1	CGAGGTGATAGTGTGGTTTATGG	GCACCATTCAACTCCTCGCTTTC
STAT6	CGCAGTTCAACAAGGAGATCC	TCCAGGACACCATCAAACCA
STEAP1	GGCAATACTGGCTCTGTTGGCT	GCGTGTATTGTGCCCAGTAGAAG
TCF4	CTTCCTCCAAACCAGCAACC	CCCAACATTCCTGCATAGCC

Gene	Forward	Reverse
TGFB3	CGAGTGGCTGTCCTTTGATG	TCTCCATTGGGCTGAAAGGT
TSPAN13	TCGCCATGTGCTCCAATCATAGG	CTGTAGGTCAGCCAAACACCCA
WNT3A	GCCCCACTCGGATACTTCTT	AGGAATACTGTGGCCCAACA
WNT5B	CTGTTCACGGCTGCTCTG	AAACATCTCGGGTCTCTGCA
ZEB1	CAGGGAGGAGCAGTGAAAGA	ACATCCTGCTTCATCTGCCT
B-ACTIN	CCAACCGCGAGAAGATGA	CCAGAGGCGTACAGGGATAG
HPRT	CAGACTTTGCTTTCCTTGGTCA	ACTTCGTGGGGTCCTTTTCA
TBP	AGTTCTGGGATTGTACCGCA	TCCTCATGATTACCGCAGCA

Table S4. Primary and secondary antibodies used for western blotting in this publication. All primary antibodies are monoclonal. 1. ab = primary antibody, 2. ab = secondary antibody

	Type	Name	Source	Catalog nr	RRID	Dilution, Buffer	2. ab (nr)
1	1. ab	Anti-β-Actin	Sigma Aldrich	A5441	AB_476744	1:20,000, BSA+TBS-0.075%T	9
2	1. ab	E-Cadherin (24E10)	Cell signaling	3195	AB_2291471	1:100, TBS-0.1%T	8
3	1. ab	N-Cadherin (13A9)	Cell signaling	14215	AB_2798427	1:500, Milk+TBS-0.1%T	9
4	1. ab	NONO	Invitrogen (Thermo Fisher)	MA3-2024	AB_2152170	1:500, Milk+TBS-0.075%T	8
5	1. ab	Notch1 (D1E11) XP(tm)	Cell signaling	3608S	AB_2153354	1:1,000, BSA+TBS-0.075%T	9
6	1. ab	PCNA	Abcam	Ab29	AB_303394	1:5,000, BSA+TBS-0.075%T	9
7	1. ab	Snail (C15D3)	Cell signaling	3879	AB_2255011	1:500, BSA+TBS-0.1%T	8
8	2. ab	Anti-rabbit IgG	Cell signaling	7074S	AB_2099233	1:5,000, Buffer of 1. ab	NA
9	2. ab	Anti-mouse IgG	Cell signaling	7076S	AB_330924	1:5,000, Buffer of 1. ab	NA

Supplementary results

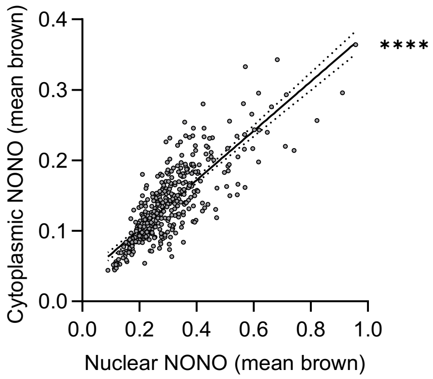

*Figure S1. Significant correlation between cytoplasmic and nuclear expression of NONO. The p-values of the simple linear regression are indicated as **** = p<0.0001.*

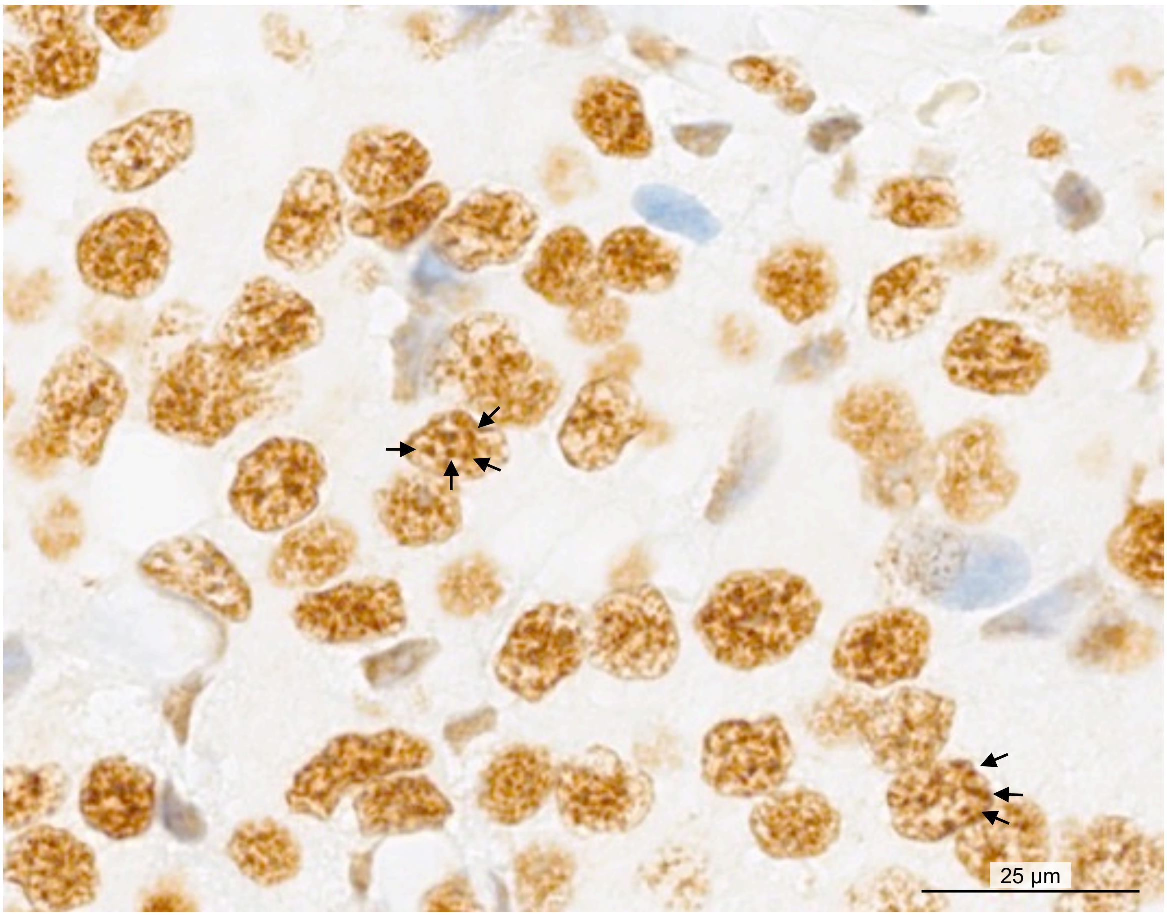

Figure S2. Representative immunohistochemistry image showing punctate nuclear NONO expression in a high expressing distant metastasis.

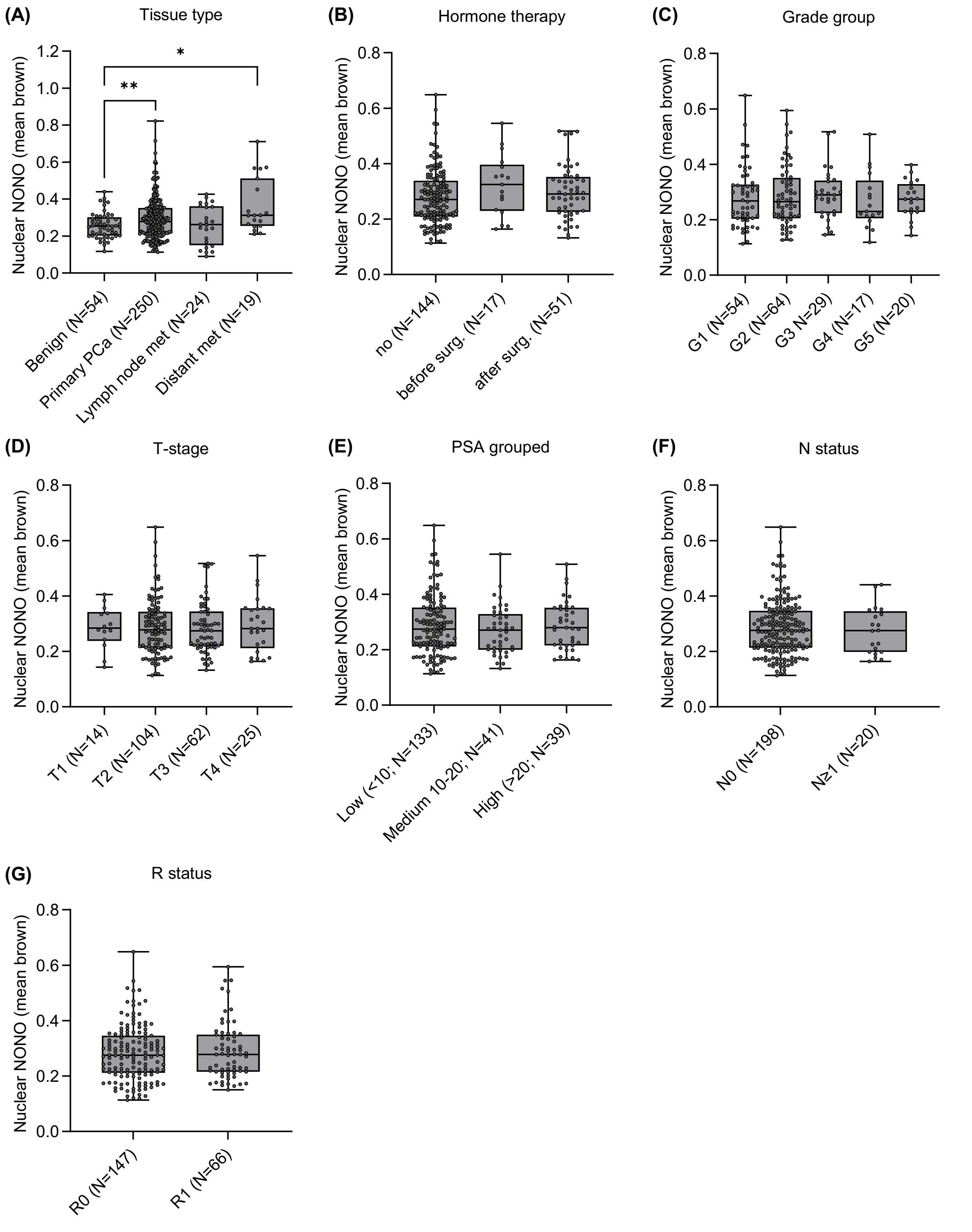

*Figure S3. Nuclear NONO expression in relation to patient and tumor characteristics. (A) Higher nuclear NONO expression in distant metastases (met) than in lymph node metastases. P's were calculated using Welch's ANOVA and the Games-Howell post-hoc test. No significant difference in NONO expression with respect to hormone therapy (B), grade group (C), T-stage (D), and grouped PSA values (E). P was calculated using Fisher's ANOVA with Tukey post-hoc test. In addition, no significant difference in NONO expression with respect to N (F) and R status (G). P was calculated using students' t test or Fisher's ANOVA and the Turkey post-hoc test depending on the number of variables. * = p < 0.05, ** = p < 0.01.*

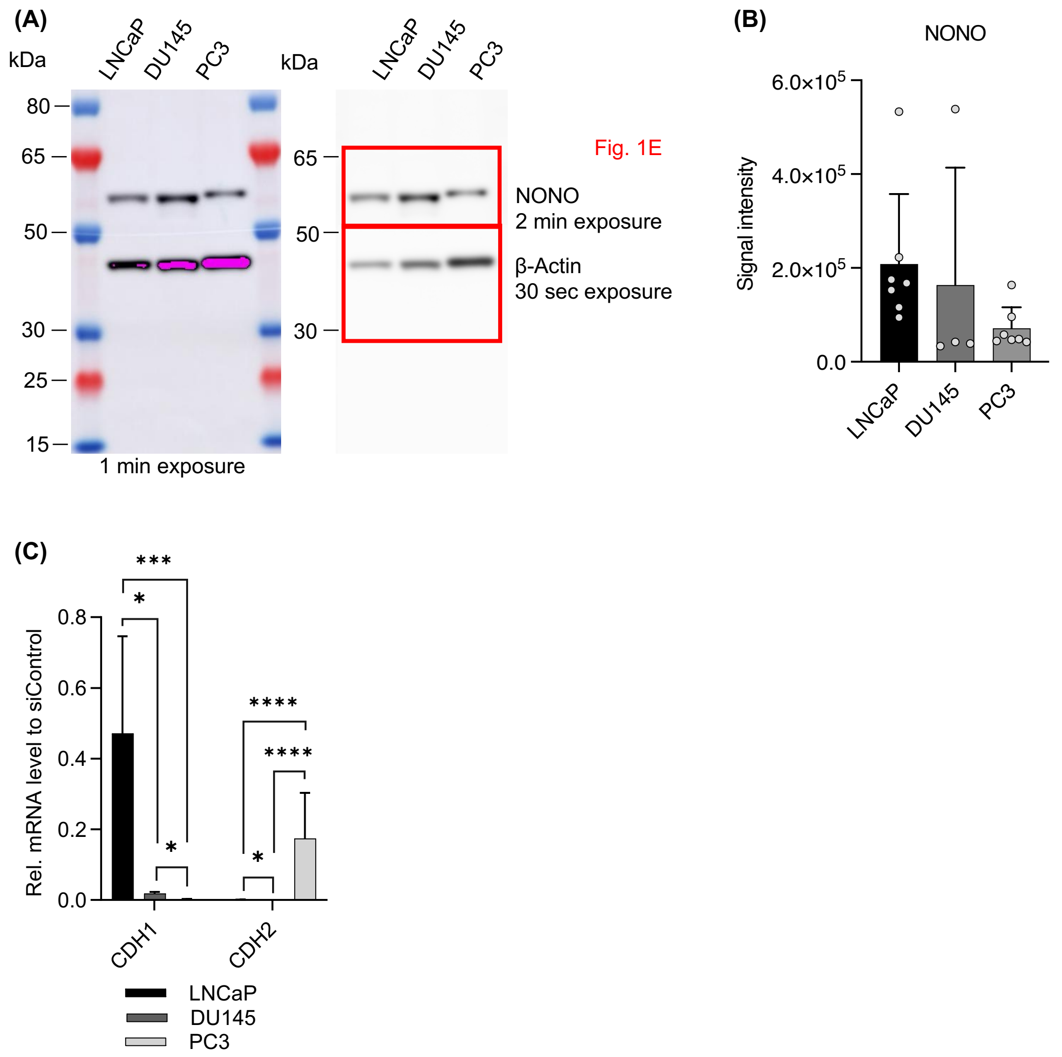

*Figure S4. The NONO expression was determined in the PCa metastasis cell lines LNCaP, DU145, and PC3 by western blot (A-B). Representative original western blot image (A) and quantification of the western blot (B) of NONO protein level. β-Actin was used as a loading control (n = 4). (C) Increased epithelial-to-mesenchymal transition in PC3 cells. The CDH1 and CDH2 expression was determined in the PCa metastasis cell lines LNCaP, DU145, and PC3 by qRT-PCR. The PCa lymph node metastasis cell line LNCaP shows the highest CDH1 expression, while the PCa bone metastasis cell line PC3 shows the highest CDH2 expression. The PCa brain metastasis cell line DU145 shows intermediate CDH1 expression and the lowest CDH2 expression. mRNA levels were determined by normalization to the mean of TBP, HPRT, and β-Actin and presented as mean fold change ± 95 % CI to siControl (n = 3). * = $p < 0.05$, ** = $p < 0.01$, *** = $p < 0.001$, **** = $p < 0.0001$.*

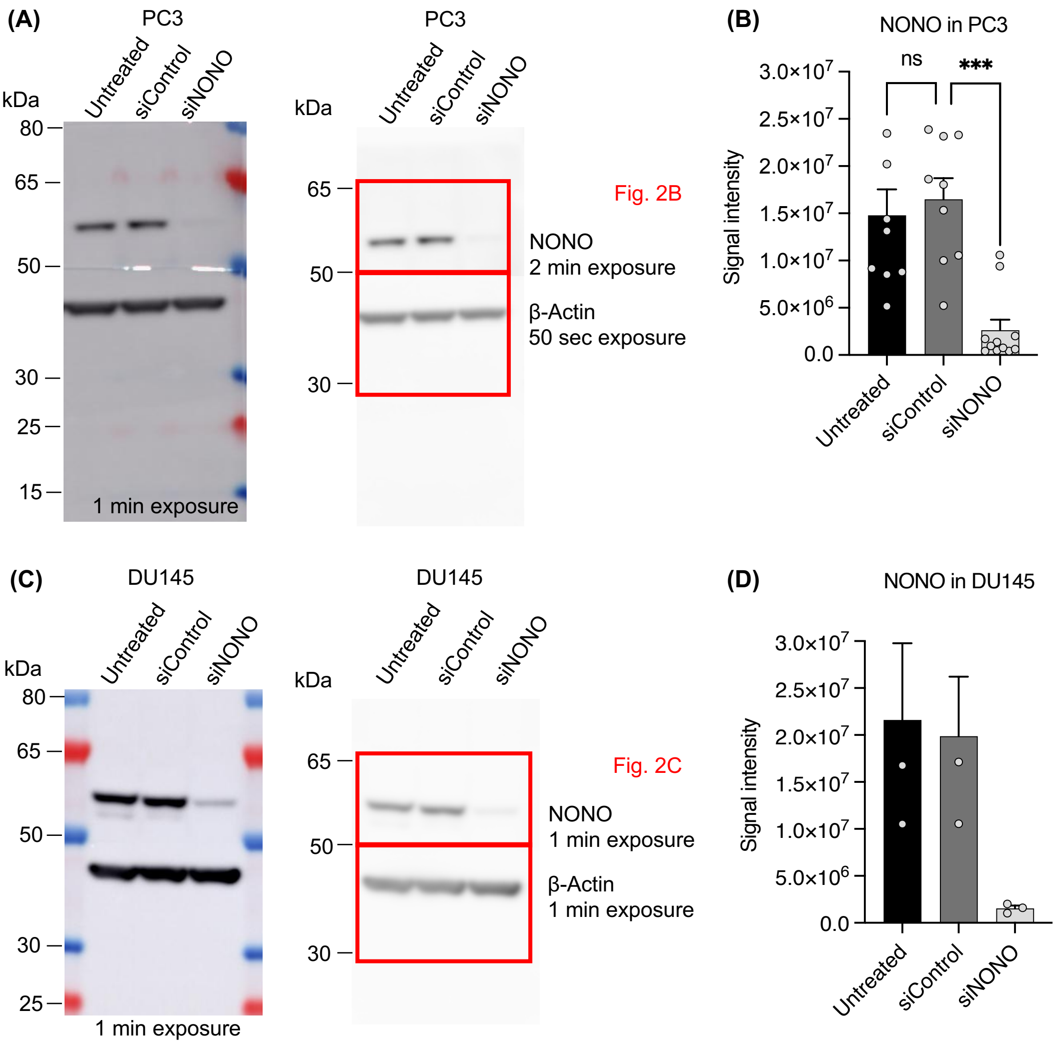

*Figure S5. NONO protein expression after siRNA-transfection in PC3 (A and B) and DU145 cells (C and D). Representative original western blot image (A, C) and quantification of the western blot (B, D) after siRNA-transfection targeting NONO. β-Actin was used as a loading control (n = 9 for transfection in PC3 cells and n = 3 for transfection in DU145 cells). *** = p < 0.001*

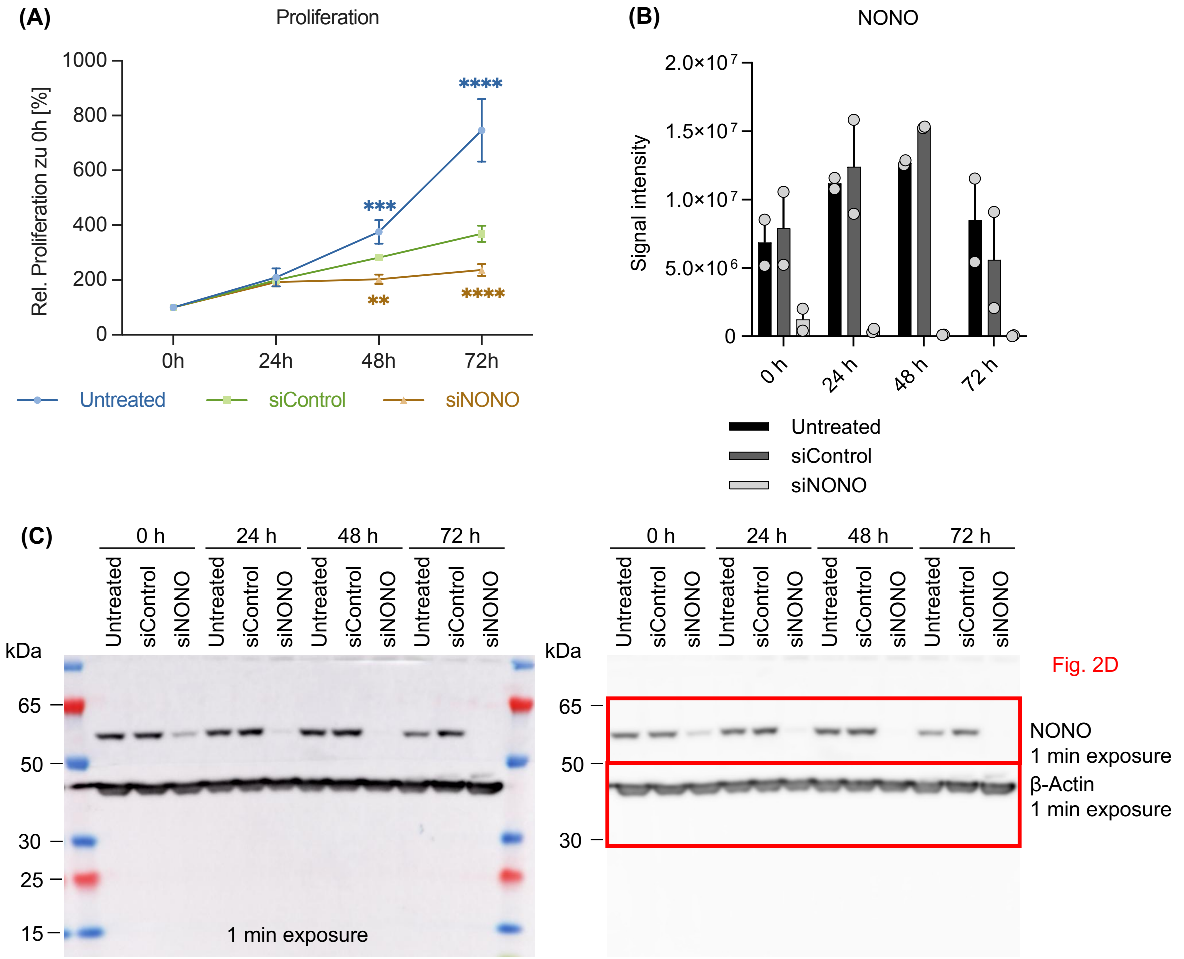

*Figure S6. (A) Inhibitory effects of siRNAs targeting NONO on the cell proliferation of PC3 cells analyzed by MTT assay. Results are shown as mean % ± 95 % CI to 0 h at 100 % (n = 3). (B-C) Stability of the knockdown efficiency of siNONO at different time points after transfection in PC3 cells was analyzed by Western blotting. Representative original western blot quantification (B) and western blot image (C) at different time points. PC3 cells were incubated with siRNA targeting NONO for 48 h. Then, transfected cells were cultured for 0 h, 24 h, 48 h and 72 h without siRNA. β-Actin was used as a loading control (n = 2). Error bars indicate the standard error of the mean (SEM). ** = p < 0.01, *** = p < 0.001, **** = p < 0.0001*

Table S5. Expression of genes related to the cell cycle. Data were obtained using RT^2 profiler PCR array for human cell cycle. Fold change (FC) in comparison to siControl. SD = standard deviation.

	RT2 Profiler PCR array human EMT						
	Untreated			siNONO			
	FC	SD	p-value	FC	SD	p-value	N
ABL1	0,90	0,141	0,4207	0,99	0,108	0,7364	4
ANAPC2	1,04	0,272	0,9959	0,78	0,035	0,0736	4
ATM	0,90	0,177	0,3095	0,85	0,045	0,0793	4
ATR	0,84	0,146	0,4909	0,64	0,059	0,1010	4
AURKA	1,11	0,264	0,8151	0,60	0,177	0,0014	4
AURKB	1,23	0,492	0,7902	0,47	0,226	0,0112	4
BCCIP	0,97	0,130	0,8939	0,51	0,148	0,0003	4
BCL2	1,17	0,587	0,9434	0,73	0,479	0,4240	4
BIRC5	1,14	0,170	0,7574	0,58	0,262	0,0094	4
BRCA1	0,99	0,124	0,9952	0,62	0,261	0,0314	4
BRCA2	1,03	0,182	0,9908	0,83	0,198	0,2465	4
CASP3	0,87	0,057	0,1816	0,65	0,020	0,0017	4
CCNA2	1,13	0,147	0,7256	0,57	0,212	0,0132	4
CCNB1	1,16	0,232	0,5302	0,63	0,243	0,0031	4

	RT2 Profiler PCR array human EMT						
	Untreated			siNONO			
	FC	SD	p-value	FC	SD	p-value	N
CCNB2	1,13	0,210	0,5702	0,66	0,279	0,0213	4
CCNC	0,83	0,125	0,0602	1,00	0,046	0,9247	4
CCND1	0,85	0,085	0,3728	1,31	0,234	0,0905	4
CCND2	1,57	0,837	0,5630	1,63	0,465	0,6761	4
CCND3	0,85	0,079	0,3161	0,91	0,163	0,7809	4
CCNE1	1,55	1,106	0,7131	0,77	0,675	0,4482	4
CCNF	0,88	0,019	0,5263	0,59	0,211	0,0060	4
CCNG1	0,84	0,133	0,2370	1,06	0,080	0,8868	4
CCNG2	0,86	0,129	0,8361	0,85	0,070	0,7974	4
CCNH	0,83	0,118	0,0356	0,78	0,123	0,0288	4
CCNT1	1,22	0,385	0,5750	1,19	0,260	0,9686	4
CDC16	1,00	0,401	0,9637	1,02	0,203	0,9465	4
CDC20	1,27	0,254	0,3944	0,55	0,177	0,0050	4
CDC25A	0,74	0,070	0,2963	0,74	0,263	0,1834	4
CDC25C	0,93	0,085	0,7652	0,51	0,215	0,0056	4
CDC34	0,88	0,212	0,5494	1,23	0,131	0,3996	4
CDC6	0,90	0,024	0,7791	0,51	0,065	0,0115	4
CDK1	1,54	0,708	0,4224	0,77	0,136	0,6118	4
CDK2	0,99	0,105	0,9955	0,44	0,035	0,0006	4
CDK4	0,87	0,088	0,2907	0,75	0,143	0,0146	4
CDK5R1	0,81	0,146	0,3428	0,84	0,183	0,2193	4
CDK5RAP1	0,73	0,209	0,1814	0,57	0,118	0,0476	4
CDK6	1,11	0,311	0,7673	1,17	0,274	0,8307	4
CDK7	0,78	0,017	0,0013	0,81	0,072	0,0122	4
CDK8	1,19	0,531	0,7159	1,38	0,050	0,0662	4
CDKN1A	0,73	0,168	0,2752	0,51	0,208	0,0206	4
CDKN1B	0,75	0,218	0,2290	1,05	0,167	0,8155	4
CDKN2A	0,84	0,097	0,3694	1,20	0,074	0,3774	4
CDKN2B	0,77	0,177	0,5447	1,47	0,370	0,5824	4
CHEK1	1,00	0,118	0,9997	0,99	0,296	0,9342	4
CHEK2	0,85	0,105	0,0733	0,81	0,228	0,0695	4
CKS1B	1,06	0,141	0,6410	0,87	0,245	0,0952	4
CKS2	0,83	0,155	0,2988	0,63	0,163	0,0024	4
CUL1	1,18	0,248	0,3938	1,12	0,215	0,7487	4
CUL2	0,92	0,136	0,4569	0,82	0,041	0,0128	4
CUL3	0,97	0,139	0,8078	1,01	0,139	0,9254	4
E2F1	0,92	0,108	0,8181	0,58	0,212	0,0281	4
E2F4	0,91	0,185	0,7075	1,01	0,276	0,7075	4
GADD45A	1,42	0,662	0,5083	1,04	0,316	0,9876	4
GTSE1	1,14	0,301	0,6945	0,11	0,032	<0,0001	4
HUS1	1,22	0,365	0,3454	1,02	0,229	0,8962	4
KNTC1	0,90	0,081	0,4071	0,68	0,196	0,0101	4
KPNA2	1,08	0,302	0,8652	0,74	0,245	0,0060	4
MAD2L1	1,10	0,184	0,8014	0,54	0,183	0,0062	4
MAD2L2	0,95	0,373	0,8036	0,59	0,189	0,1047	4
MCM2	1,28	0,158	0,3941	0,77	0,131	0,0920	4
MCM3	0,99	0,115	0,9934	0,80	0,180	0,1085	4
MCM4	0,96	0,143	0,9562	0,67	0,175	0,1252	4
MDM2	0,82	0,090	0,2197	0,86	0,047	0,4582	4
MKI67	1,15	0,241	0,5687	0,52	0,222	0,0068	4
MNAT1	0,93	0,143	0,5267	0,87	0,084	0,1721	4
MRE11A	1,25	0,607	0,7377	1,02	0,304	0,9986	4
NBN	0,94	0,136	0,6817	0,88	0,025	0,3138	4
RAD1	0,91	0,119	0,4985	0,77	0,078	0,0436	4
RAD17	0,99	0,152	0,9586	0,99	0,061	0,8132	4
RAD51	0,77	0,084	0,4444	0,55	0,168	0,0987	4
RAD9A	1,27	0,191	0,1093	1,09	0,160	0,8971	4
RB1	1,02	0,176	0,9936	0,98	0,177	0,9950	4
RBBP8	0,98	0,066	0,9845	1,00	0,363	0,9948	4
RBL1	1,13	0,326	0,8117	0,88	0,214	0,4515	4
RBL2	0,91	0,166	0,5665	1,06	0,108	0,7611	4
SERTAD1	0,86	0,077	0,5600	1,10	0,279	0,5705	4

	RT2 Profiler PCR array human EMT						
	Untreated			siNONO			
	FC	SD	p-value	FC	SD	p-value	N
SKP2	0,87	0,171	0,5973	1,07	0,231	0,9740	4
STMN1	2,67	2,759	0,3948	1,75	1,291	0,7267	4
TFDP1	1,14	0,223	0,6499	0,35	0,053	0,0006	4
TFDP2	0,78	0,019	0,2140	0,68	0,188	0,0405	4
TP53	0,86	0,133	0,5224	1,20	0,222	0,2434	4
WEE1	0,74	0,137	0,0335	0,83	0,272	0,3553	4

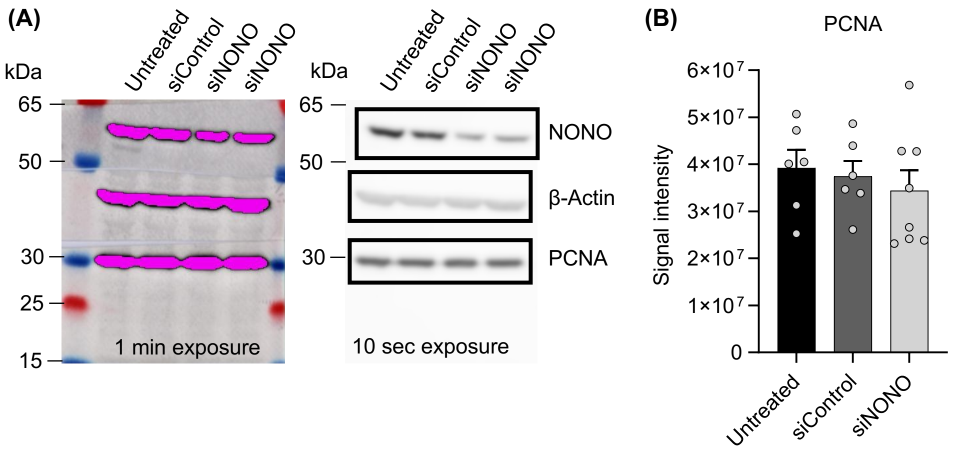

Figure S7. PCNA protein expression in siRNA-transfected PC3 cells targeting NONO. Representative original western blot image (A) and quantification of the western blot (B). β-Actin was used as a loading control (n = 6). Error bars indicate the standard error of the mean (SEM).

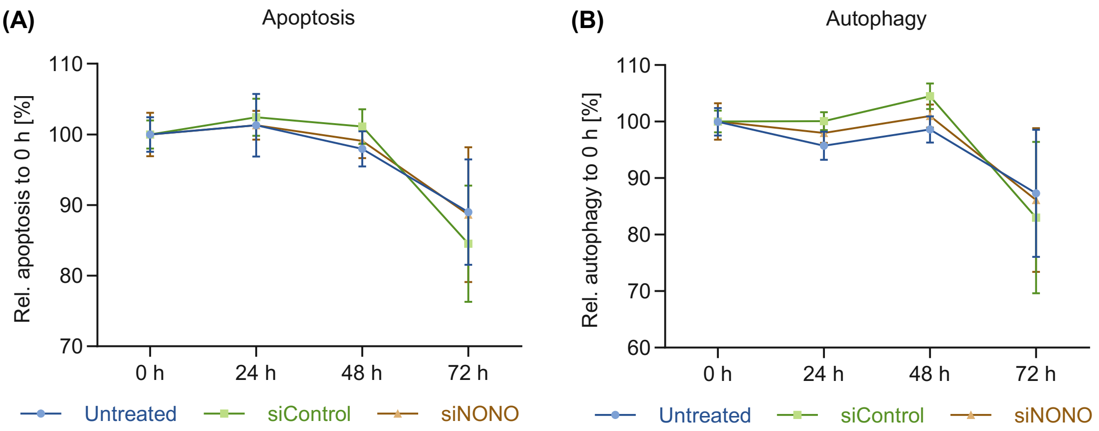

Figure S8. Apoptotic (A) and autophagic (B) behavior at different time points. PC3 cells were incubated with siRNA targeting NONO for 48 h. Then, transfected cells were cultured for 0 h, 24 h, 48 h and 72 h without siRNA and apoptosis and autophagy was measured. Results are shown as mean % ± 95 % CI to 0 h at 100 % (n = 2).

Table S6. Expression of genes related to the epithelial to mesenchymal transition (EMT). Data were obtained RT^2 profiler PCR array for human EMT and/or using real-time quantitative PCR (qRT-PCR). Fold change (FC) in comparison to siControl. SD = standard deviation.

	RT2 Profiler PCR array human EMT							qPCR						
	Untreated			siNONO				Untreated			siNONO			
	FC	SD	p-value	FC	SD	p-value	N	FC	SD	p-value	FC	SD	p-value	N
AHNAK	0,58	0,524	0,4918	0,93	0,267	0,4541	3							
AKT1	0,91	0,766	0,6792	0,90	0,254	0,3867	3							

	RT2 Profiler PCR array human EMT							qPCR						
	Untreated			siNONO				Untreated			siNONO			
	FC	SD	p-value	FC	SD	p-value	N	FC	SD	p-value	FC	SD	p-value	N
AXIN2								0,77	0,262	0,1241	1,03	0,308	0,9976	4
BMP1	0,55	0,464	0,5009	1,40	0,252	0,9028	3							
BMP2	0,57	0,474	0,4919	0,80	0,269	0,1702	3							
BMP7	2,35	3,409	0,9027	5,20	7,354	0,9964	3	1,51	0,612	0,3092	1,27	0,343	0,6163	4
CALD1	0,72	0,604	0,5765	1,21	0,210	0,9973	3							
CAMK2N1	0,64	0,610	0,5278	1,18	0,118	>0,9999	3							
CAV2	0,86	0,773	0,6417	1,18	0,676	0,7924	3							
CDH1	0,64	0,613	0,5524	1,45	0,344	0,8935	3	1,04	0,483	0,9669	1,14	0,333	0,8738	6
CDH2	0,78	0,656	0,5933	0,93	0,209	0,3706	3	0,96	0,088	0,9629	1,00	0,205	0,9887	6
COL1A2	0,78	0,670	0,6385	2,27	1,367	0,1377	3	2,03	0,920	0,1398	2,17	1,578	0,2731	6
COL3A1	0,62	0,548	0,5142	1,57	0,348	0,5090	3	1,10	0,230	0,9653	1,34	0,491	0,8793	3
COL5A2	0,68	0,644	0,5754	1,23	0,182	0,9829	3							
CTNNB1	0,83	0,797	0,6247	1,11	0,477	0,9260	3							
DESI1	0,77	0,643	0,6415	1,15	0,148	0,9944	3							
DSC2	0,68	0,567	0,6294	1,26	0,163	0,9647	3							
DSP	0,61	0,522	0,5165	1,22	0,039	0,9660	3							
EGFR	0,72	0,648	0,5977	0,98	0,057	0,3550	3							
ERBB3	0,71	0,623	0,5811	1,31	0,102	0,9559	3							
ESR1	0,96	0,424	0,9936	7,10	10,157	0,6095	3	0,86	0,515	0,5038	0,70	0,261	0,3600	6
F11R	0,71	0,600	0,5933	1,49	0,331	0,3449	3	1,05	0,173	0,9710	1,32	0,175	0,1154	3
FGFBP1	0,58	0,550	0,4870	0,64	0,123	0,2092	3	1,16	0,414	0,9106	0,52	0,145	0,0458	5
FN1	0,66	0,616	0,5347	1,40	0,018	0,4130	3	0,89	0,230	0,9027	1,13	0,063	0,6313	2
FOXC2	0,93	0,826	0,6999	2,33	2,120	0,0890	3	0,86	0,284	0,6174	0,75	0,307	0,2648	3
FZD7	0,60	0,514	0,5096	0,98	0,168	0,6235	3							
GEMIN2	0,72	0,678	0,5815	1,33	0,385	0,8222	3							
GLI1								1,49	0,684	0,5580	3,22	1,743	0,0403	5
GNG11	0,95	0,825	0,7272	2,07	0,146	0,0608	3	1,17	0,292	0,8383	2,12	0,584	0,0240	5
GSC	0,95	0,503	0,8544	3,91	3,582	0,1906	3							
GSK3B	0,80	0,702	0,6471	1,14	0,073	0,9735	3							
HHIP								0,95	0,090	0,8031	0,46	0,052	0,0008	4
IGFBP4	0,64	0,531	0,5347	0,89	0,168	0,3636	3	0,91	0,209	0,7983	0,73	0,179	0,2531	4
IL1RN	0,38	0,316	0,3414	1,61	0,386	0,2850	3	0,83	0,412	0,6186	1,38	0,551	0,6209	5
ILK	0,79	0,691	0,6359	1,21	0,082	0,8955	3							
ITGA5	0,58	0,483	0,8955	0,81	0,041	0,0100	3	0,77	0,217	0,2132	0,87	0,484	0,4999	4
ITGAV	0,94	0,829	0,6732	2,07	0,573	0,6732	3	0,87	0,115	0,5880	1,05	0,315	0,9992	3
ITGB1	0,70	0,606	0,6732	1,46	0,178	0,4945	3							
JAG1	0,62	0,674	0,4945	0,91	0,219	0,4945	3	0,94	0,083	0,7407	0,67	0,125	0,0341	6
KDM5C								0,91	0,284	0,9435	0,92	0,182	0,9651	5
KRT14	0,44	0,184	0,4945	1,95	1,982	0,4945	3							
KRT19	0,66	0,597	0,5117	0,64	0,246	0,1988	3	0,98	0,253	0,9927	0,54	0,089	0,3672	4
KRT7	0,46	0,492	0,4270	1,14	0,861	0,8931	3	0,93	0,265	0,9350	1,35	0,400	0,6011	4
MAP1B	0,75	0,703	0,6336	1,61	0,299	0,1985	3	0,89	0,234	0,4353	1,32	0,099	0,0578	4
MMP2	0,17	0,136	0,1320	2,67	1,494	0,9400	3	0,98	0,441	0,7482	1,07	0,411	0,9996	5
MMP3	1,49	1,473	0,8803	3,29	0,074	0,0034	3	1,35	0,361	0,6984	2,78	1,168	0,0205	5
MMP9	0,83	0,746	0,6598	1,13	0,131	0,8887	3							
MSN	0,84	0,696	0,6726	1,17	0,217	0,9807	3							
MST1R	0,89	0,769	0,6679	1,28	0,411	0,9540	3							
NONO								1,04	0,273	0,9995	0,05	0,024	0,0001	7
NOTCH1	0,48	0,452	0,3813	0,66	0,053	0,0042	3	1,12	0,224	0,7263	0,56	0,106	0,0125	6
NUDT13	0,36	0,305	0,3353	0,99	0,307	0,6699	3							
OCLN	0,83	0,809	0,6144	1,71	0,344	0,1529	3	1,18	0,745	0,9994	1,23	0,391	0,9855	4
PDGFRB	0,74	0,599	0,6921	0,47	0,348	0,0553	3							
PLEK2	0,97	0,783	0,7869	1,15	0,200	0,7554	3							
PTK2	0,69	0,571	0,5901	1,19	0,047	0,9956	3							
PTP4A1	0,74	0,686	0,5962	1,25	0,172	0,6187	3							
RAC1	0,71	0,642	0,6036	0,78	0,145	0,0313	3	1,02	0,108	0,9949	0,74	0,061	0,2250	4
RGS2	0,70	0,576	0,6149	0,86	0,054	0,2887	3							
SERPINE1	0,53	0,452	0,4635	1,11	0,055	0,7562	3							
SHH								3,71	2,282	0,5249	2,27	0,915	0,7468	4
SMAD2	0,68	0,602	0,5794	1,40	0,109	0,2319	3	0,99	0,119	0,9697	1,02	0,055	0,9313	3
SMAD4								1,14	0,150	0,4599	0,90	0,062	0,5216	3
SNAI1	0,60	0,506	0,5792	0,94	0,126	0,6850	3	0,97	0,190	0,9453	1,06	0,426	0,9961	6

	RT2 Profiler PCR array human EMT							qPCR						
	Untreated			siNONO				Untreated			siNONO			
	FC	SD	p-value	FC	SD	p-value	N	FC	SD	p-value	FC	SD	p-value	N
SNAI2	0,78	0,710	0,6275	1,20	0,313	0,9057	3	1,04	0,164	0,9076	0,87	0,241	0,1911	6
SNAI3	0,52	0,428	0,4220	1,12	0,048	0,6951	3							
SOX10	0,83	0,641	0,8161	2,85	2,315	0,9912	3	1,49	0,737	0,8060	0,66	0,081	0,6196	5
SPARC	0,62	0,530	0,6286	1,40	0,273	0,9675	3	0,56	0,289	0,5033	0,64	0,192	0,6702	4
SPP1	0,29	0,263	0,4712	0,68	0,777	0,5896	3	1,51	0,601	0,3078	2,99	1,139	0,0151	5
STAT3	0,77	0,744	0,6181	0,99	0,280	0,9740								
STAT6								1,14	0,133	0,6565	1,06	0,223	0,9450	4
STEAP1	0,67	0,542	0,5938	1,48	0,214	0,0526	3	0,90	0,174	0,5186	1,12	0,205	0,6983	4
TCF3	0,77	0,680	0,6378	0,84	0,159	0,2513	3							
TCF4	0,70	0,590	0,6035	1,65	0,117	0,0580	3	1,01	0,072	0,9958	1,26	0,070	0,0884	4
TFPI2	0,80	0,711	0,6243	1,12	0,205	0,9973	3							
TGFB1	0,57	0,501	0,4963	1,05	0,021	0,9912	3							
TGFB2	0,63	0,680	0,5000	1,20	0,249	0,8747	3							
TGFB3	0,76	0,647	0,6714	1,92	0,360	0,0276	3	1,03	0,194	0,9982	1,45	0,240	0,2342	5
TIMP1	0,57	0,470	0,5643	1,05	0,201	0,8952	3							
TMEFF1	0,87	0,701	0,7297	1,19	0,257	0,9665	3							
TMEM132A	0,64	0,531	0,5225	1,28	0,293	0,9917	3							
TSPAN13	0,80	0,663	0,6241	0,58	0,093	0,0157	3	1,09	0,163	0,8585	0,52	0,073	0,0073	5
TWIST1	0,58	0,483	0,5057	1,11	0,097	0,8794	3							
VCAN	0,87	0,755	0,7200	2,11	0,138	0,2286	3							
VIM	0,81	0,694	0,6525	1,00	0,224	0,6535	3							
VPS13A	0,59	0,544	0,5221	1,29	0,327	0,7701	3							
WNT11	0,58	0,494	0,6714	1,53	1,678	0,9956	3							
WNT3A								1,16	0,551	0,9996	0,74	0,591	0,4046	4
WNT5A	0,63	0,548	0,5017	1,25	0,119	0,4811	3							
WNT5B	1,04	1,054	0,9376	3,13	3,626	0,7450	3	0,79	0,315	0,5964	0,82	0,314	0,8175	4
ZEB1	0,83	0,682	0,7486	1,56	0,552	0,2985	3	1,09	0,227	0,8699	1,04	0,043	0,9630	4
ZEB2	0,65	0,541	0,5696	1,07	0,119	0,9521	3							

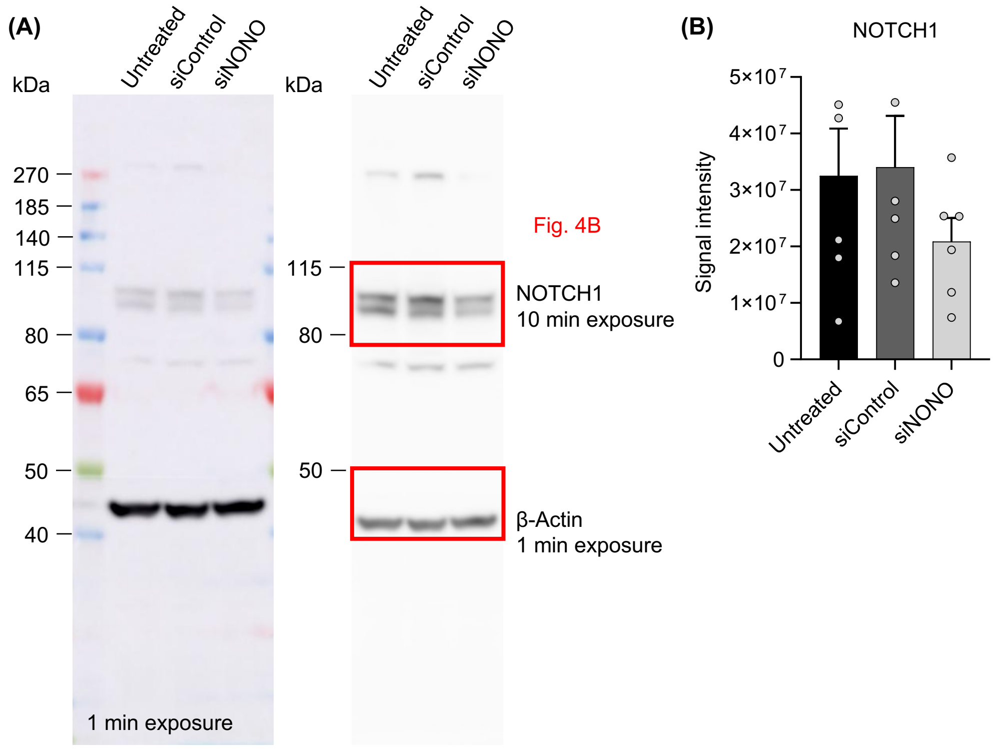

Figure S9. NOTCH1 protein expression in siRNA-transfected PC3 cells targeting NONO. Representative original western blot image (A) and quantification of the western blot (B). β-Actin was used as a loading control (n = 6). Error bars indicate the standard error of the mean (SEM).

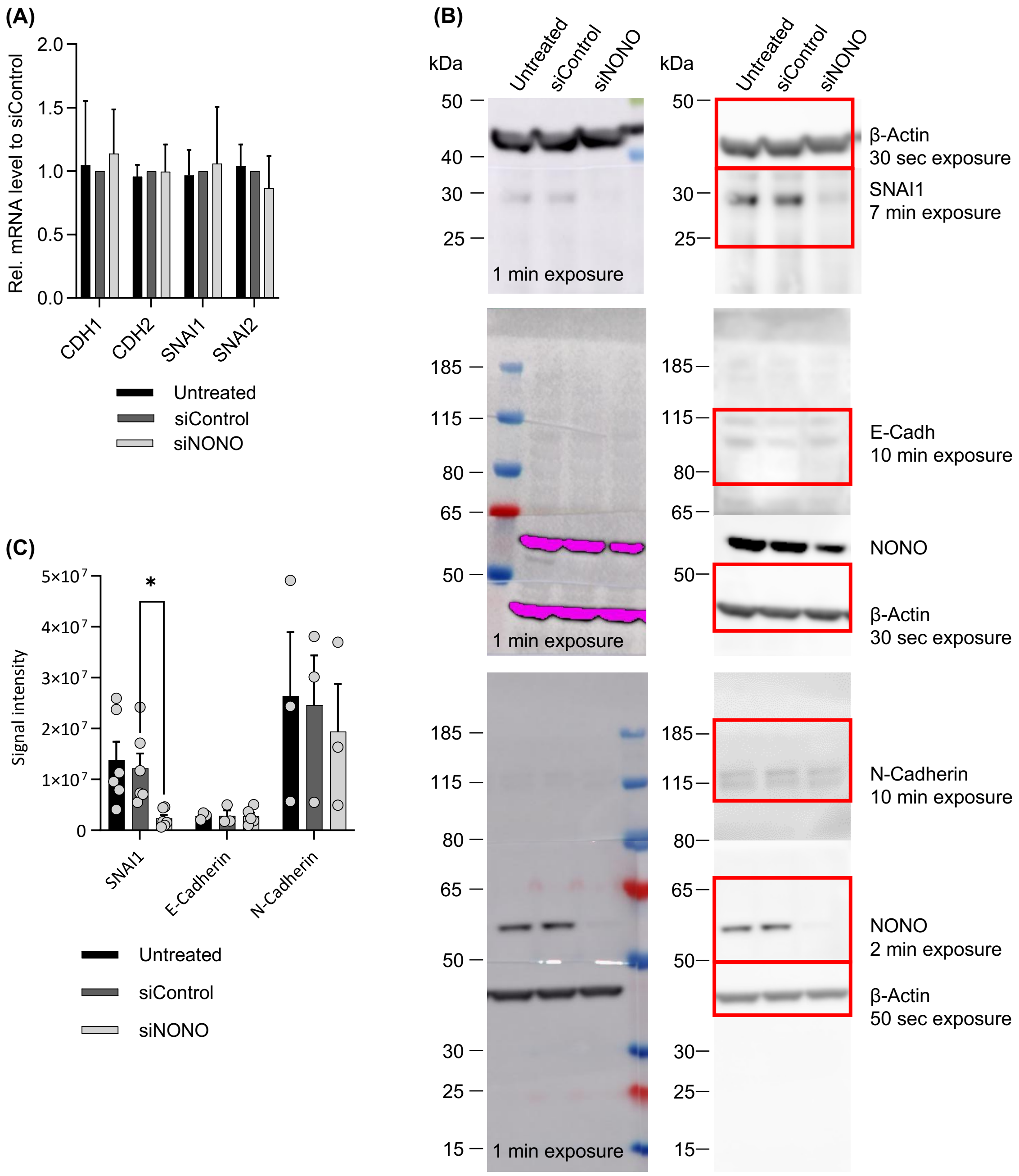

Figure S10. Knockdown of NONO led to slight changes in SNAI1 protein expression but showed no differences in other EMT markers. (A) siRNA transfection targeting NONO showed no effect on the mRNA expression of CDH1 and CDH2 in PC3 cells. Analysis was performed using qRT-PCR. mRNA levels were determined by normalization to the mean of TBP, HPRT, and β-Actin and presented as mean fold change ± 95 % CI to siControl (n = 6). (B) Knockdown of NONO in PC3 cells reduced the protein level of SNAI1 but not the level of E-Cadherin and N-Cadherin compared to the siControl. The protein level was analyzed using Western blotting. β-Actin was used as a loading control (n = 6 for SNAI1 and n = 3 for E-Cadherin and N-Cadherin). (C) Quantification of the western blot. Error bars indicate the standard error of the mean (SEM).
